# Disproportionality analysis of interstitial lung disease associated with novel antineoplastic agents during breast cancer treatment: a pharmacovigilance study

**DOI:** 10.1016/j.eclinm.2025.103160

**Published:** 2025-03-18

**Authors:** Zijun Zhu, Yongxin Li, Chaoyong Zhu, Qiuxia Dong, Yixiao Zhang, Zhilin Liu, Dengfeng Ren, Fuxing Zhao, Jiuda Zhao

**Affiliations:** a Breast Disease Diagnosis and Treatment Centre, Affiliated Hospital of Qinghai University, Affiliated Cancer Hospital of Qinghai University, Xining, China; b Department of Oncology, Qinghai Red Cross Hospital, Xining, Qinghai, China; c Breast and Thyroid Surgery Department, Affiliated Hospital of Qinghai University, Affiliated Cancer Hospital of Qinghai University, Xining, China

**Keywords:** Adverse event, Breast cancer, Interstitial lung disease, Novel antineoplastic agent

## Abstract

**Background:**

Studies have shown that some antineoplastic agents may be associated with interstitial lung disease (ILD), but large-scale real-world data are lacking. This study aimed to detect signals of disproportionate reporting for ILD associated with novel antineoplastic agents used in breast cancer treatment.

**Methods:**

In this pharmacovigilance study, we collected data from the FDA Adverse Event Reporting System (FAERS; Jan 01, 2004–Dec 31, 2023) and the Japanese Adverse Drug Event Report (JADER; Jan 01, 2004–Mar 31, 2024) databases. Data retrieval involved direct download of structured datasets from the FDA and PMDA portals. Participant selection included reports of FDA-approved novel antineoplastic agents for breast cancer with documented ILD as a preferred term, excluding duplicates, non-breast cancer indications, unapproved drugs, and cases where drugs were classified as concomitant or interacting. Signals of disproportionate reporting were assessed using the reporting odds ratio (ROR), with statistical significance defined as a lower 95% confidence interval >1 and ≥3 ILD cases.

**Findings:**

A total of 2913 patients with ILD from FAERS and 1868 from JADER were analysed. We identified 9 agents with reporting signals for ILD in FAERS: ROR and 95% confidence interval (CI) for trastuzumab deruxtecan was 12.17 (95% CI 11.04–13.41), atezolizumab 6.04 (5.02–7.28), everolimus 3.21 (2.95–3.50), abemaciclib 2.87 (2.52–3.27), pertuzumab 2.84 (2.49–3.25), olaparib 2.29 (1.65–3.19), trastuzumab emtansine 2.27 (1.91–2.69), pembrolizumab 2.06 (1.65–2.58), and trastuzumab 1.36 (1.25–1.49). 7 drugs associated with ILD in JADER are also captured in FAERS. Fatal cases presented with a shorter median onset time compared to nonfatal cases (56 vs. 71 days in FAERS, *P* = 0.015; 59 vs. 76.5 days in JADER, *P* = 0.046). Analyses indicated stronger reporting associations between novel antineoplastic agents and ILD compared to chemotherapeutics (FAERS: OR 2.47, 2.16–2.81; JADER: OR 1.61, 1.37–1.88; *P* < 0.0001). ILD reports were more frequent among older patients (FAERS: HR 1.0097, 1.0036–1.0159, *P* = 0.0020; JADER: HR 1.0183, 1.0094–1.0270, *P* < 0.0001), while higher weight correlated with fewer reports (FAERS: HR 0.9783, 0.9729–0.9836; *P* < 0.0001).

**Interpretation:**

Our study detected signals of disproportionate reporting for ILD with some novel antineoplastic agents in breast cancer, fatal cases had a shorter median onset time than nonfatal ones. Novel antineoplastic agents showed stronger signal of disproportionate reporting associations with ILD than chemotherapeutics. Older age and lower weight were associated with more frequent ILD reports. The limitations—including incomplete data, inherent pharmacovigilance biases, and coprescription bias—preclude causal interpretation of the observed associations and may lead to overestimation or underestimation of reporting signals. These findings highlight the need for vigilant ILD monitoring but require validation through prospective studies to clarify true clinical risks.

**Funding:**

None.

**Research in context**

**Evidence before this study**

We searched PubMed, Embase, and the Cochrane Library for studies on interstitial lung disease (ILD) associated with novel antineoplastic agents used in breast cancer treatment, without language restrictions, with the search date up to April 30, 2024. The search terms included “interstitial lung disease”, “pneumonitis”, “novel antineoplastic agents”, “breast cancer”, “tyrosine kinase inhibitors”, “monoclonal antibodies”, “antibody-drug conjugates”, “immune checkpoint inhibitors”, “CDK4/6 inhibitors”, “PARP inhibitors” and “PI3K/AKT/mTOR inhibitors”. Existing evidence indicates that antineoplastic drugs are the primary cause of drug-induced interstitial lung disease (DIILD), accounting for 23%–51% of cases and certain novel antineoplastic agents are associated with ILD, with varying incidences reported in clinical trials and case reports. However, most studies focus on individual drugs or specific classes, lacking comprehensive real-world analyses comparing ILD cases across different novel agents in patients with breast cancer. Additionally, randomised controlled trials (RCTs) may not fully capture the true incidence and severity of ILD due to strict inclusion criteria and limited follow-up durations.

**Added value of this study**

This study provides the first large-scale real-world analysis of ILD associated with a broad range of novel antineoplastic agents in breast cancer treatment, using data from the FDA Adverse Event Reporting System (FAERS) and the Japanese Adverse Drug Event Report (JADER) database. By analyzing a large and diverse patient population, we identified that multiple novel antineoplastic agents, particularly monoclonal antibodies, antibody-drug conjugates, immune checkpoint inhibitors, and mTOR inhibitors, showed signals of disproportionate reporting associated with ILD. Furthermore, by sensitivity analysis, we found that 5 novel antineoplastic agents, T-Dxd, abemaciclib, everolimus, T-DM1, and pertuzumab, showed signal of disproportionate reporting in both databases. Simultaneously, analyses suggested stronger reporting associations between novel agents and ILD compared to chemotherapeutics. We analysed the time to onset and identified potential factors associated with ILD. Our findings offer a comprehensive overview of ILD signals of disproportionate reporting associated with these agents in real-world clinical practice.

**Implications of all the available evidence**

Our study highlights the signals of disproportionate reporting of ILD associated with novel antineoplastic agents in breast cancer treatment, emphasizing the need for vigilant monitoring and early intervention to manage ILD. Clinicians should be aware that different agents have varying correlations with ILD and consider patient-specific factors such as age and weight when prescribing these therapies. The findings can inform clinical decision-making, help clinicians promptly identify and manage ILD, thereby improving patients' treatment persistence and quality of life. However, this study has limitations inherent to pharmacovigilance analyses. Residual biases, particularly coprescription bias from unmeasured drug combinations, may persist due to incomplete clinical data and reporting selectivity. Spontaneous reports enable signal detection but lack causal inference, and missing dose data precluded dose-ILD analysis. Cross-database deduplication risks misclassification due to limited matching criteria. Underreporting and surveillance biases may further limit signal completeness. These limitations necessitate validation through prospective studies or real-world evidence.

## Introduction

Novel antineoplastic agents have demonstrated outstanding efficacy in breast cancer therapy.^1^, ^2^, ^3^, ^4^, ^5^, ^6^, ^7^ However, with their widespread use, including tyrosine kinase inhibitors (TKIs), monoclonal antibodies (mAbs), antibody-drug conjugates (ADCs), immune checkpoint inhibitors (ICIs), cyclin-dependent kinase 4/6 (CDK4/6) inhibitors, poly ADP-ribose polymerase (PARP) inhibitors, and phosphatidylinositol 3-kinase (PI3K)/protein kinase B (AKT)/mammalian target of rapamycin (mTOR) inhibitors, a spectrum of adverse drug reactions (ADRs) associated with them has garnered increasing attention. Among these ADRs, interstitial lung disease (ILD), a diverse group of diseases affecting the pulmonary parenchyma, may interrupt treatment, compromise patients' quality of life, and even result in mortality. The clinical manifestations of ILD are complex and variable, ranging from acute onset with dyspnea and fever to worsening dyspnea and pulmonary dysfunction in chronic progression.^8^ Due to the increasing number of reports of drug-related ILDs in recent years, it has become one of the most scrutinized adverse reactions and has been classified as an Important Medical Event (IME) by the European Medicines Agency (EMA), emphasizing the urgent need for continuous monitoring and focused research.^9^

The incidence of ILD associated with conventional chemotherapeutic agents in breast cancer treatment is relatively low and has not received adequate attention in the past.^10^ However, currently, novel antineoplastic agents are recognized as a major cause of drug-related ILD.^11^ ADCs, ICIs, mTOR inhibitors, and EGFR-targeted inhibitors (TKIs and mAbs) exhibit a higher incidence of ILD.^12^^,^^13^ Despite limited data on drug-associated ILD with PARP inhibitors and CDK4/6 inhibitors, there are relevant pharmacovigilance analyses and case reports documenting instances of ILD, some of which were fatal.^14^, ^15^, ^16^, ^17^

Randomised controlled trials (RCTs) employ strict criteria and specific drug dosages, which may not fully capture the true incidence of ILD. In contrast, real-world studies typically have longer follow-up time that allow for the detection of later-stage ILD, providing a more accurate reflection for clinical occurrence.^18^ For instance, a pooled analysis of RCTs on trastuzumab deruxtecan (T-Dxd) found a 15.5% ILD incidence, with 2.4% fatal outcomes, while a recent real-world study found that 22% developed ILD and 5% had fatal events, a proportion significantly higher than RCT.^19^^,^^20^ Therefore, understanding the real-world occurrence of ILD with novel antineoplastic agents is crucial for clinical practice.

Individual Case Safety Report (ICSR) databases provide valuable real-world data through case safety reports, enabling the identification of adverse event signals that may not be captured in RCTs, especially for ILD. Disproportionality analysis, effective for large and heterogeneous patient populations, helps detect disproportional reporting signals for ILD, offering a more comprehensive understanding of drug safety in real-world settings.

Previous studies have mainly focused on individual drugs or those within the same class, and lacked comparisons across different classes of antineoplastic agents.^21^, ^22^, ^23^, ^24^, ^25^ In contrast, our study is the first large-scale real-world analysis evaluating ILD signals across novel antineoplastic agents approved for breast cancer. By utilizing two large adverse reaction databases, we provide a comprehensive analysis of ILD cases across multiple drug classes in patients with breast cancer, offering new insights into the comparative safety profile of these agents.

Here, we conduct a large sample size study aimed to detect signals of disproportionate reporting for ILD associated with novel antineoplastic agents in breast cancer, thereby identifying drugs that may be associated with ILD. Meanwhile, we explored potential factors influencing the occurrence of ILD.^26^

## Methods

### Data source

This disproportionality analysis is based on the FDA Adverse Event Reporting System (FAERS) and Japanese Adverse Drug Event Report (JADER) databases. Managed by the FDA, FAERS is a comprehensive database that gathers spontaneous reports of adverse events and medication errors from healthcare professionals, consumers, and manufacturers. The seven different types of datasets include in the FAERS data files: patient demographic and administrative information (DEMO), drug information (DRUG), adverse events (REAC), patient outcomes (OUTC), report sources (RPSR), therapy start dates and end dates for reported drugs (THER), and indications for drug administration (INDI). JADER, managed by the Pharmaceuticals and Medical Devices Agency (PMDA) of Japan, functions similarly to FAERS, gathering reports of adverse drug reactions from healthcare professionals, pharmaceutical companies, and consumers. It comprises four datasets: DEMO, DRUG, REAC, and primary disease (HIST). In both databases, all adverse events are coded using the preferred term (PT) according to the international Medical Dictionary for Regulatory Activities (MedDRA) (Version 26.1). FAERS and JADER are publicly available pharmacovigilance databases containing fully anonymized and de-identified patient records. Therefore, ethics approval and informed consent were exempted for this study.

### Procedures

We collected data on FDA-approved novel antineoplastic agents used in the treatment of breast cancer from FAERS database for the period spanning Jan 01, 2004 to Dec 31, 2023, and in JADER from Jan 01, 2004 to Mar 31, 2024, including TKIs (lapatinib, neratinib, tucatinib), mAbs (trastuzumab, pertuzumab), ADCs (trastuzumab emtansine, trastuzumab deruxtecan, sacituzumab govitecan), ICIs (pembrolizumab, atezolizumab, avelumab), CDK4/6 inhibitors (palbociclib, ribociclib, abemaciclib), PARP inhibitors (olaparib, niraparib, talazoparib) and PI3K/AKT/mTOR inhibitors (alpelisib, everolimus, capivasertib), we also took into account the trade names of these drugs. To address duplicate reports, we followed FDA recommendations for data deduplication. Cases with the same “primaryid” were identified as duplicates, and only the most complete report was retained. In cases where multiple reports shared identical demographic or clinical information (e.g., age, gender, adverse event type, and event onset date), the most recent or most complete entry was included in the dataset. In general, four patterns of drug reports are identified in FAERS: “primary suspect”, “secondary suspect”, “concomitant”, and “interacting”. Three categories are recognized in JADER: “suspected medicine”, “concomitant medicine”, and “interaction”. For precise results, only ADR reports with roles categorized as “primary suspect/suspected medicine” were included. Furthermore, we used the narrow “ILD” standardized MedDRA query (SMQ) to precisely identify PTs associated with ILD (SMQ code: 20000042). Data were collected based on all PTs included in the narrow SMQ of ILD, with a complete list of these PTs provided in the eTable 1. At the same time, to avoid the potential impact of certain PTs in the SMQ that were not associated with cancer therapy, we performed additional sensitivity analyses with “interstitial lung disease” as the only PT for case selection. To identify reports associated with breast cancer, we included cases containing “breast cancer” in the “indication” field of the FAERS and JADER databases. Reports with unspecified or unrelated indications were excluded. For cases with ambiguous or missing indications, we cross-referenced the approved indications of the study drugs at the time of reporting to confirm their relevance to breast cancer treatment. We excluded duplicate cases from both databases based on gender, age, drug, event date, and country of reporting in FAERS, additional disproportionate analyses were performed on the JADER database. To ensure methodological transparency and reproducibility, this study adhered to the READUS-PV guideline.^27^^,^^28^

### Statistical analysis

We conducted a disproportionality analysis using the reporting odds ratio (ROR) to assess the potential association between novel antineoplastic agents in treatment of breast cancer and the occurrence of ILD. To be considered statistically significant and indicative of a potential signal, the lower limit of the 95% confidence interval (CI) for the ROR (ROR_025_) >1, and there should be at least 3 case reports of ILD.^29^ The formulas for calculating the ROR and the 95% CI are provided in the Appendix. Upon identifying signals, we descriptively analysed the clinical characteristics of the cases. The period from therapy start to the occurrence of ILD events was analysed as the time to onset (TTO), using median, quartiles, and the Weibull shape parameter (WSP) to describe its distribution. Scale (alpha) and shape (beta) are the two parameters that describe the Weibull distribution. The scale parameter alpha defines the scale of the distribution, indicating the spread of time to onset across the dataset. The shape parameter beta determines the distribution's shape, influencing how the hazard changes over time: when beta less than 1 (95% CI < 1) indicates a decreasing hazard rate (early failure type), around 1 (95% CI includes 1) suggests a constant hazard rate (random failure type), and greater than 1 (95% CI > 1) indicates an increasing hazard rate (wear-out failure type). We used the Kruskal–Wallis test to compare ILD TTO among different drugs and the Wilcoxon rank-sum test to compare the TTO of the same drug across two databases. The Pearson chi-square test was utilized to compare mortality rates across various drugs or populations. *P* < 0.05 indicates statistical significance. We applied univariate logistic regression to assess the association between drug class (novel antineoplastic agents vs. chemotherapeutic agents) and the development of ILD. The odds ratios (OR) and corresponding 95% CI were calculated for each drug class. Moreover, volcano plots were created to visually compare the signals of ILD between novel antineoplastic agents and chemotherapeutic agents. This plot was generated using the ROR along with the adjusted *P* obtained from Fisher's exact test followed by Bonferroni correction, allowing for a clear visualization of the differences in ILD signal between the two drug classes. Univariate Cox models evaluated the individual impact of each drug category on ILD, while a multivariate model assessed the effects of clinical characteristics. We additionally conducted a disproportionate analysis of ILD cases reported by healthcare professionals to further validate the reliability of the results. All statistical analyses were performed using R (version 4.3.1) and RStudio programs.

To reduce potential biases and improve the robustness of the results, this study employed a multivariable logistic regression model to analyse the association between novel antineoplastic agents exposure and the occurrence of ILD.^30^ Covariates were adjusted to estimate the adjusted RORs (aRORs), and 95% CIs. Reports with missing data on key covariates were excluded to ensure the validity of the multivariable logistic regression analysis. We also included concomitant drugs that were most common and likely to cause ILD as confounding variables in logistic regression models along with other relevant variables. To evaluate the sensitivity and specificity of the disproportionality analysis using ROR, external positive and negative control drugs were introduced. Positive control drug (amiodarone) was selected based on established evidence of a significant association with ILD in previous studies, while negative control drug (letrozole) was chosen based on a lack of evidence linking them to ILD. Disproportionality analysis was conducted using the FAERS database, and the ROR and 95% CI were calculated for each control drug. Sensitivity was assessed by confirming that positive control drugs produced significant positive association signals (ROR025 > 1), while specificity was evaluated by verifying that negative control drugs yielded no significant association signals (95% CI includes 1).

### Role of the funding source

There was no funding source for this study. All authors had full access to the data in the study. JZ had final responsibility for the decision to submit for publication.

## Results

### Descriptive analysis

**Fig. 1:**
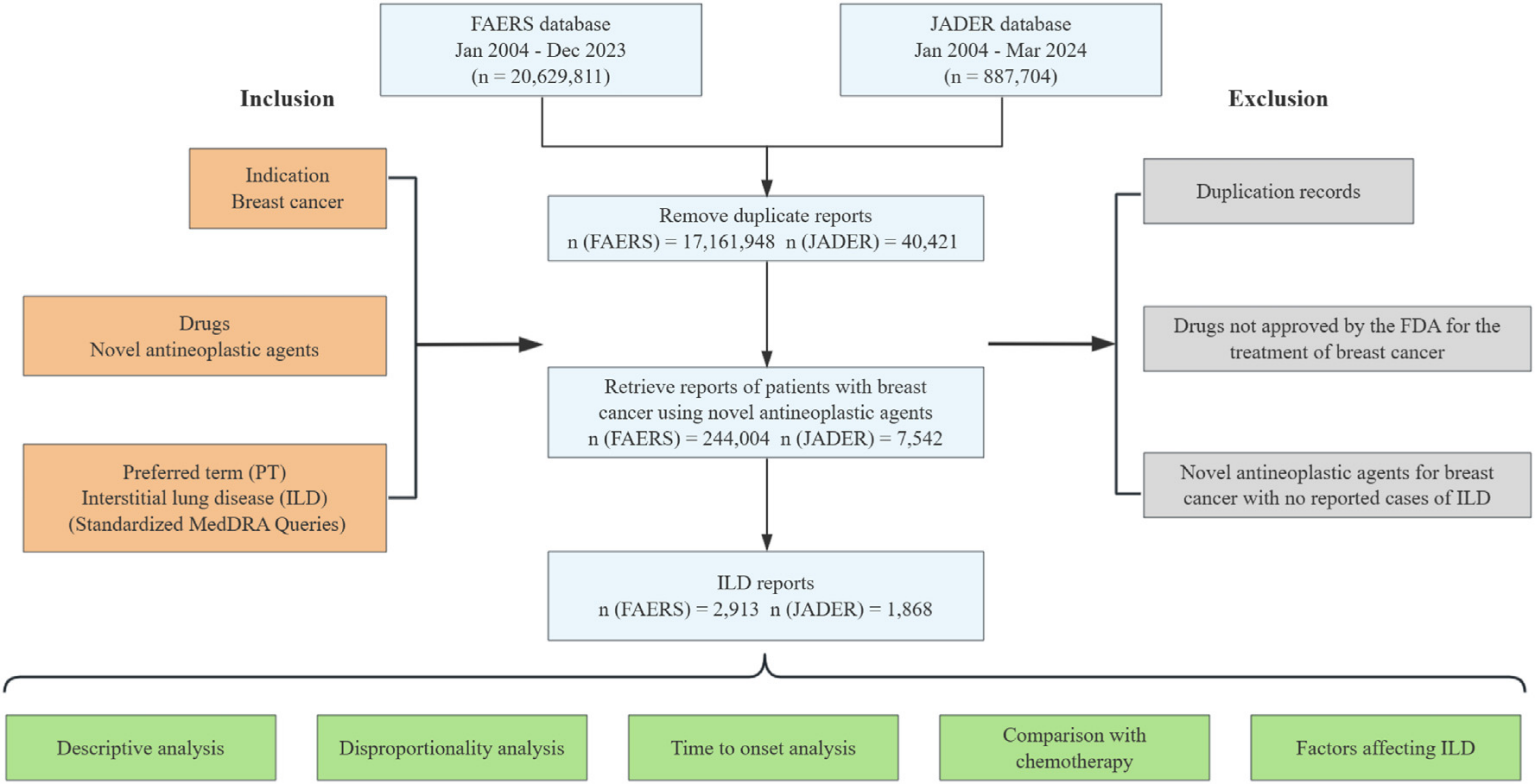
**Flow chart of the study** Abbreviations: FAERS, FDA Adverse Event Reporting System; JADER, Japanese Adverse Drug Event Report; MedDRA, Medical Dictionary for Regulatory Activities.

In FAERS, a total of 2913 cases and 3175 reports of ILD were identified. The median age was 64 years (interquartile range [IQR] 55–71). The median weight was 63 kg (IQR: 53–75). Among the 2551 cases with available outcomes, more than half (67.86%, 1731) involved severe outcomes, including death (20.97%, 535), life-threatening (6.47%, 165), disability (0.94%, 24), and other serious (39.47%, 1007). Similarly, we identified 1868 cases of ILD and 1894 related reports in JADER. Most of them were over 60 years (67.46%, 937/1389), predominantly within the range of 40–59 kg (66.20%, 470/710), and 8.82% cases (134/1519) were fatal (Table 1). The flow of data processing is detailed in Fig. 1.

**Table 1:** Clinical characteristics of patients with breast cancer-associated interstitial lung disease in the FAERS and JADER databases.

Characteristics	FAERS No. (%)	JADER No. (%)
Number of patients	2913	1868
Gender		
Data available	2681	1822
Female	2654 (98.99%)	1801 (98.85%)
Male	27 (1.01%)	21 (1.15%)
Age (years)		
Data available	1811	1389
<30	3 (0.17%)	1 (0.07%)
30–39	85 (4.69%)	23 (1.66%)
40–49	187 (10.33%)	106 (7.63%)
50–59	408 (22.53%)	322 (23.18%)
60–69	581 (32.08%)	499 (35.93%)
70–79	424 (23.41%)	354 (25.49%)
≥80	123 (6.79%)	84 (6.05%)
Median (IQR)	64 (55–71)	NA
Weight (kg)		
Data available	1110	710
<40	30 (2.70%)	55 (7.75%)
40–49	155 (13.96%)	244 (34.37%)
50–59	280 (25.23%)	226 (31.83%)
60–69	244 (21.98%)	129 (18.17%)
70–79	199 (17.93%)	47 (6.62%)
80–89	113 (10.18%)	NA
≥80	NA	9 (1.27%)
≥90	89 (8.02%)	NA
Median (IQR)	63 (53–75)	NA
Reported countries (Top 5)		
United States	939 (32.23%)	NA
Japan	619 (21.25%)	NA
Germany	189 (6.49%)	NA
France	136 (4.67%)	NA
Canada	107 (3.67%)	NA
Outcomes		
Data available	2551	1519
Death	535 (20.97%)	134 (8.82%)
Life-threatening	165 (6.47%)	NA
Hospitalization	818 (32.07%)	NA
Disability	24 (0.94%)	NA
Required intervention	2 (0.08%)	NA
Other serious	1007 (39.47%)	NA
Recovery	NA	558 (36.73%)
Remission	NA	601 (39.57%)
Unrecovered	NA	202 (13.30%)
With aftereffect	NA	24 (1.58%)

Note: Some characteristics are only reported in one of the databases (FAERS or JADER), resulting in NA for these categories in the other database.

Abbreviations: FAERS, FDA Adverse Event Reporting System; JADER, Japanese Adverse Drug Event Report; IQR, interquartile range; NA, not applicable.

### Disproportionality analysis

**Fig. 2:**
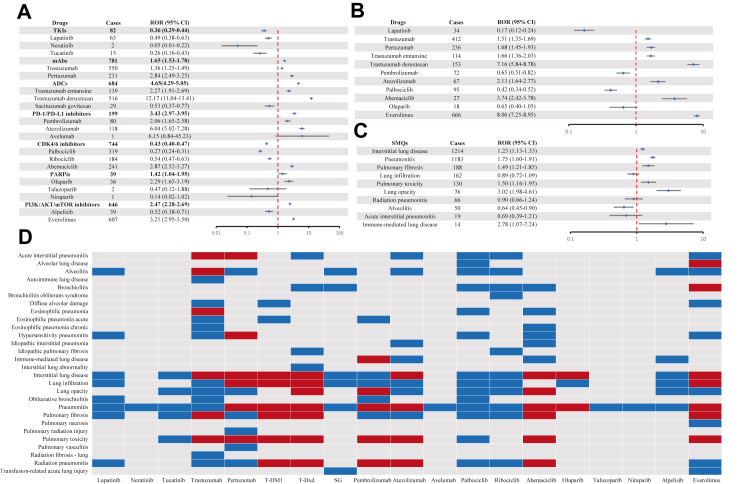
**The signal of ILD associated with novel antineoplastic agents in the treatment of breast cancer**. (A) Forest plot of ROR values for different novel antineoplastic agents associated with ILD in the FAERS database. (B) Forest plot of ROR values for different novel antineoplastic agents associated with ILD in the JADER database (C) ROR values of the top 10 most frequently reported PTs under the SMQ level for ILD in the FAERS database. (D) Heatmap showing the signal of disproportionate reporting for the PT level in the FAERS database among different novel antineoplastic agents. Red indicates that the lower limit of the 95% CI of the ROR >1 and at least 3 cases of ILD were reported. Blue indicates that the lower limit of the 95% CI of the ROR <1, or fewer than 3 cases of ILD were reported. Gray indicates no ILD-related reports. Abbreviations: ROR, reporting odds ratio; CI, confidence interval; TKIs, tyrosine kinase inhibitors; mAbs, monoclonal antibodies; ADCs, antibody-drug conjugates; T-DM1, trastuzumab emtansine; T-Dxd, trastuzumab deruxtecan; SG, sacituzumab govitecan; ICIs, immune checkpoint inhibitors; CDK4/6, cyclin-dependent kinase 4/6; PARP, poly ADP-ribose polymerase; and PI3K, phosphatidylinositol 3-kinase; AKT, protein kinase B; mTOR, mammalian target of rapamycin; SMQ, standardized MedDRA query; ILD, interstitial lung disease; FAERS, FDA Adverse Event Reporting System; JADER, Japanese Adverse Drug Event Report; PT, preferred term.

In FAERS, 9 novel antineoplastic agents were identified as signal of disproportionate reporting for ILD (Fig. 2A): T-Dxd with the ROR of 12.17 (95% CI 11.04–13.41), atezolizumab (ROR 6.04; 95% CI 5.02–7.28), everolimus (ROR 3.21; 95% CI 2.95–3.50), abemaciclib (ROR 2.87; 95% CI 2.52–3.27), pertuzumab (ROR 2.84; 95% CI 2.49–3.25), olaparib (ROR 2.29; 95% CI 1.65–3.19), trastuzumab emtansine (T-DM1) (ROR 2.27; 95% CI 1.91–2.69), pembrolizumab (ROR 2.06; 95% CI 1.65–2.58), and trastuzumab (ROR 1.36; 95% CI 1.25–1.49). Furthermore, at the PT level, 49 positive signals were identified across various drugs, Fig. 2D presents the PTs of ILD events associated with novel antineoplastic agents. T-Dxd and everolimus led with the most signals, each presenting 7. Following closely were trastuzumab, pertuzumab, T-DM1, and abemaciclib, each with 6. Pembrolizumab reported 5 signals, atezolizumab 4, and olaparib with 2. The results in the univariate Cox analysis were highly consistent presented by the ROR, ADCs (HR 2.83; 95% CI 2.39–3.35), ICIs (HR 2.17; 95% CI 1.72–2.72) and PI3K/AKT/mTOR inhibitors (HR 2.84; 95% CI 2.46–3.28) were associated with a higher odds of reporting for ILD compared to other novel antineoplastic agents. In contrast, TKIs (HR 0.57; 95% CI 0.42–0.76) and CDK4/6 inhibitors (HR 0.34; 95% CI 0.30–0.39) were associated with a lower odds of reporting for ILD. Although neither mAbs (HR 1.04; 95% CI 0.90–1.20) nor PARP inhibitors (HR 1.42; 95% CI 0.87–2.33) showed a statistically significant difference in odds of reporting for ILD, it is important to consider that the Cox model compares different novel antineoplastic agents, which are primary contributors to ILD. Thus, the relevance of these drugs to ILD remains non-negligible, even if the difference is not statistically significant (eFigures 1 and 2). In JADER, 7 drugs were identified as signal of disproportionate reporting for ILD (Fig. 2B): Everolimus (ROR 8.06; 95% CI 7.25–8.95), T-Dxd (ROR 7.16; 95% CI 5.84–8.78), abemaciclib (ROR 3.74; 95% CI 2.42–5.78), atezolizumab (ROR 2.13; 95% CI 1.64–2.77), pertuzumab (ROR 1.68; 95% CI 1.45–1.93), T-DM1 (ROR 1.66; 95% CI 1.36–2.03), and trastuzumab (ROR 1.51; 95% CI 1.35–1.69). Notably, pembrolizumab and olaparib exhibited ILD signals in FAERS; but not in JADER. Nevertheless, there was a high degree of concordance between both databases regarding the other drugs. Overall, monoclonal antibodies (mAbs), antibody-drug conjugates (ADCs), programmed death-1/programmed death-ligand 1 (PD-1/PD-L1) inhibitors, and mTOR inhibitors were more likely to accompany ILD. After removing duplicates from JADER and identifying 1492 cases and 1502 reports, we conducted disproportionality and sensitivity analyses and compared the results with the main analyses, details in Appendix (eTable 2). In FAERS, we analysed the top 10 most frequently reported PTs under the SMQ level to identify correlations (Fig. 2C); details in Appendix. The raw data used to calculate the ROR in the disproportionality analysis are shown in eTable 3.

### Sensitivity analysis

To validate the sensitivity and specificity of the disproportionality analysis using ROR, amiodarone and letrozole were selected as external positive and negative control drugs, respectively. Amiodarone was chosen based on its well-documented association with ILD, as it is one of the most common causes of DIILD, with reported incidence rates ranging from 1.2% to 8.8%.^12^ In contrast, letrozole was selected for its lack of evidence linking it to ILD.^31^ In patients with breast cancer, the ROR for amiodarone-associated ILD was 20.57 (95% CI: 12.39–34.51), confirming a strong positive association consistent with its established ILD signal. Conversely, the ROR for letrozole-associated ILD was 0.93 (95% CI: 0.83–1.05), indicating no association. These results support the sensitivity of the ROR method in detecting known signal of disproportionate reporting and its specificity in identifying drugs without ILD signals.

To mitigate the effect of confounding factors on ILD associated with novel antineoplastic agents, we performed multivariate logistic regression analyses, controlling for age, weight, reporter type, reporting region (without JADER), and whether or not the drug was co-administered, and calculating the aROR and its 95% CIs. In addition, to minimize the impact of other concomitant drugs that may cause ILD, we listed in eTable 4 the top 50 drugs most frequently used concomitantly. Based on FDA labelling information, we found that among these drugs, docetaxel, paclitaxel, cyclophosphamide, and vinorelbine may be associated with the occurrence of ILD. We included these four drugs as confounding variables, along with other relevant variables, in the logistic regression model. To minimize indication bias, the analysis was restricted to patients with breast cancer receiving novel antineoplastic agents. We identified 6 novel antineoplastic agents signals that may be associated with ILD after adjusting for confounding factors using multivariate logistic regression analysis in FAERS: T-Dxd (aROR 5.37; 95% CI 4.27–6.75), abemaciclib (aROR 4.97; 95% CI 3.89–6.36), everolimus (aROR 4.48; 95% CI 3.71–5.41), pertuzumab (aROR 1.67; 95% CI 1.32–2.11), T-DM1 (aROR 1.55; 95% CI 1.17–2.05), and atezolizumab (aROR 1.55; 95% CI 1.15–2.09). Similarly, in JADER, 6 novel antineoplastic agents signals were identified: T-Dxd (aROR 14.00; 95% CI 10.42–18.82), everolimus (aROR 5.16; 95% CI 3.72–7.16), abemaciclib (aROR 3.24; 95% CI 1.75–5.99), pertuzumab (aROR 1.69; 95% CI 1.22–2.35), trastuzumab (aROR 1.56; 95% CI 1.24–1.97), and T-DM1 (aROR 1.54; 95% CI 1.10–2.15) (Table 2). Notably, these agents were also identified in the pre-adjusted disproportionality analysis, indicating general consistency in the observed associations before and after adjustment.

**Table 2:** Adjusted ROR for ILD associated with novel antineoplastic agents in the treatment of breast cancer.

Drugs	aROR (95% CI)	*P* value
FAERS		
Lapatinib	0.57 (0.41–0.80)	0.0010
Neratinib	1.47 (0.20–10.82)	0.71
Tucatinib	1.03 (0.38–2.83)	0.95
Trastuzumab	0.96 (0.80–1.16)	0.68
Pertuzumab	1.67 (1.32–2.11)	<0.0001
Trastuzumab emtansine	1.55 (1.17–2.05)	0.0020
Trastuzumab deruxtecan	5.37 (4.27–6.75)	<0.0001
Sacituzumab govitecan	1.06 (0.58–1.95)	0.84
Pembrolizumab	1.19 (0.65–2.20)	0.57
Atezolizumab	1.55 (1.15–2.09)	0.0040
Avelumab	4.34 (0.55–34.09)	0.16
Palbociclib	0.45 (0.37–0.55)	<0.0001
Ribociclib	1.02 (0.75–1.38)	0.91
Abemaciclib	4.97 (3.89–6.36)	<0.0001
Olaparib	1.61 (0.58–4.49)	0.36
Talazoparib	0.40 (0.05–2.87)	0.36
Alpelisib	0.54 (0.25–1.14)	0.11
Everolimus	4.48 (3.71–5.41)	<0.0001
**JADER**		
Lapatinib	0.30 (0.19–0.48)	<0.0001
Trastuzumab	1.56 (1.24–1.97)	<0.0001
Pertuzumab	1.69 (1.22–2.35)	0.0020
Trastuzumab emtansine	1.54 (1.10–2.15)	0.012
Trastuzumab deruxtecan	14.00 (10.42–18.82)	<0.0001
Pembrolizumab	1.33 (0.61–2.90)	0.47
Atezolizumab	1.45 (0.87–2.42)	0.15
Palbociclib	0.94 (0.66–1.34)	0.74
Abemaciclib	3.24 (1.75–5.99)	<0.0001
Olaparib	0.55 (0.22–1.39)	0.21
Everolimus	5.16 (3.72–7.16)	<0.0001

Abbreviations: ROR, reporting odds ratio; ILD, interstitial lung disease; FAERS, FDA Adverse Event Reporting System; aROR, adjusted reporting odds ratio; CI, confidence interval; JADER, Japanese Adverse Drug Event Report.

In order to enhance the credibility of the results and to more accurately reflect the specific relationship between ILD and novel antineoplastic agent, we performed sensitivity analyses using ILD as the only PT for case selection. The results revealed that the same 5 drugs were identified with signal of disproportionate reporting in FAERS and JADER databases, consistently across both unadjusted and adjusted disproportionality analyses (eTable 5). In FAERS, T-Dxd [ROR 17.14 (15.04–19.55), aROR 6.88 (4.76–9.96)]; pertuzumab [ROR 4.44 (3.74–5.26), aROR 2.34 (1.59–3.46)]; abemaciclib [ROR 3.44 (2.86–4.13), aROR 5.02 (3.31–7.63)]; T-DM1 [ROR 2.21 (1.69–2.90), aROR 1.89 (1.22–2.93)]; everolimus [ROR 1.44 (1.19–1.73), aROR 1.96 (1.28–3.01)]. In JADER, everolimus [ROR 8.76 (7.88–9.75), aROR 5.48 (3.94–7.64)]; T-Dxd [ROR 6.35 (5.14–7.86), aROR 11.28 (8.39–15.16)]; abemaciclib [ROR 3.57 (2.27–5.63), aROR 3.33 (1.79–6.19)]; pertuzumab [ROR 1.76 (1.52–2.04), aROR 1.87 (1.34–2.61)]; T-DM1 [ROR 1.57 (1.27–1.94), aROR 1.51 (1.06–2.14)]. In addition to the above 5 drugs, trastuzumab was also identified as a signal of disproportionate reporting in JADER with ROR 1.59 (1.42–1.78) and aROR 1.73 (1.37–2.18).

### Time to onset analysis of ILD

**Fig. 3:**
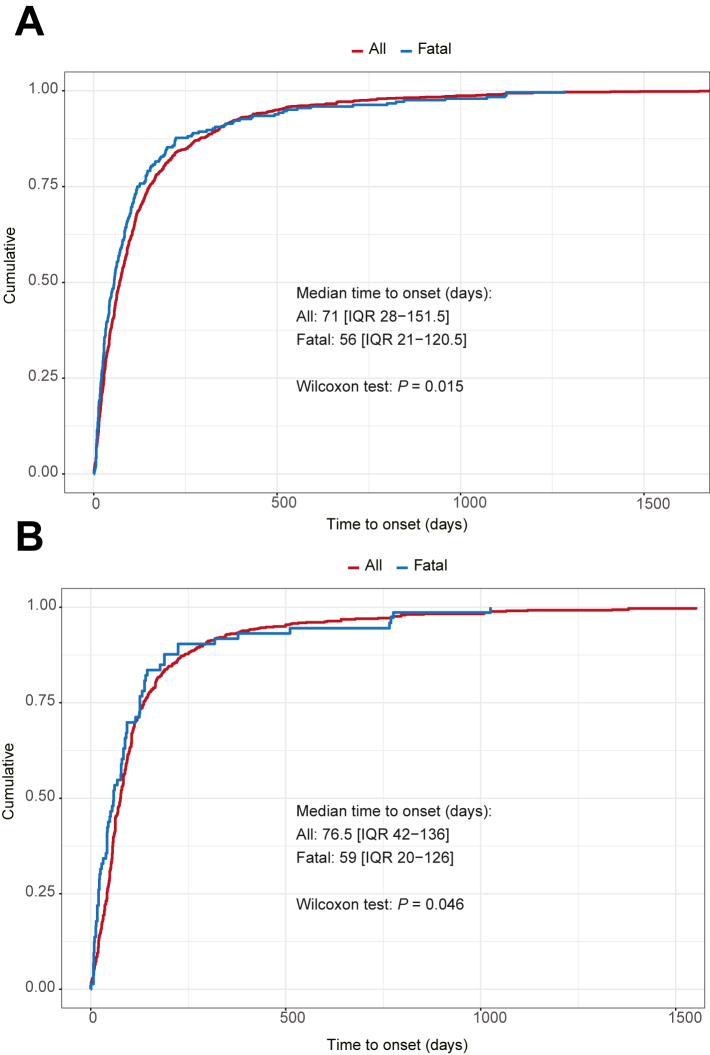
**Cumulative distribution curves showing the time to onset of ILD after treatment with novel antineoplastic agents for all cases and fatal cases in the FAERS (A) and JADER (B) databases**. Statistical significance was assessed using the nonparametric Wilcoxon rank sum test. Abbreviations: IQR, interquartile range; ILD, interstitial lung disease; FAERS, FDA Adverse Event Reporting System; JADER, Japanese Adverse Drug Event Report.

In FAERS, the median onset time of ILD associated with all novel antineoplastic agents was 71 days (IQR: 28–151.5), compared to 56 days (IQR: 21–120.5) for fatal cases, with a significant difference (*P* = 0.015) (Fig. 3A). We observed significant differences in the TTO of ILD among the various drugs. The median onset time was the shortest at 19 days (IQR: 15–250) for olaparib and the longest at 138 days (IQR: 74–323) for palbociclib (eTable 6). In JADER showed a median onset time of 76.5 days (IQR 42–136), compared to 59 days (IQR: 20–126) for fatal cases, indicating a significant difference (*P* = 0.046) (Fig. 3B). Pembrolizumab and everolimus each demonstrated the shortest median onset times for ILD at 61 days, with IQRs of 23–101 and 44–104.5, respectively, and the longest for abemaciclib at 205 days (IQR 89.8–279) (eTable 6). The results indicated that, with the exception of abemaciclib (median onset times: 57.5 days [IQR: 27.8–143] in FAERS vs. 205 days [IQR: 89.8–279] in JADER, *P* = 0.0032) and everolimus (56 days [IQR: 23.5–104.5] in FAERS vs. 61 days [IQR: 44–104.5] in JADER, *P* = 0.017), the differences in onset times for the same drug across the two databases were not statistically significant. Trastuzumab and pertuzumab exhibited a shape parameter beta <1 across both databases, indicating an early failure type with a relatively less reporting of ILD as time progresses. Palbociclib, ribociclib, and abemaciclib only showed this type in FAERS. In JADER, pembrolizumab demonstrated a shape parameter beta >1, indicating a wear-out failure with increasing ILD reporting over time. Other drugs showed a random failure type, suggesting that ILD could occur at any point during treatment without specific time dependency (eTable 6).

### Analysis of fatal cases

**Fig. 4:**
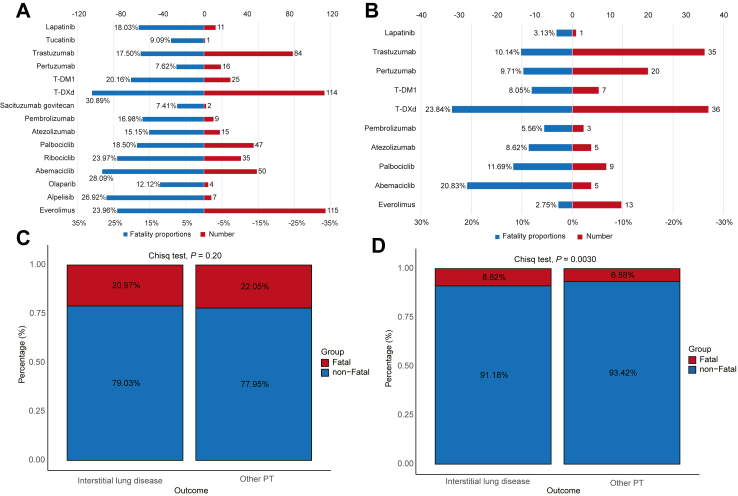
**Analysis of ILD-associated fatal cases with novel antineoplastic agents**. (A) and (B) showing the fatality proportions and number for different novel antineoplastic agents in the FAERS and JADER databases, respectively. (C) and (D) showing the comparison of fatality risk between ILD and non-ILD (other PT) patients in the FAERS and JADER databases, respectively. Statistical significance was assessed using the chi-square test. Abbreviations: T-DM1, trastuzumab emtansine; T-Dxd, trastuzumab deruxtecan; Chisq, chi-square; ILD, interstitial lung disease; PT, preferred term; FAERS, FDA Adverse Event Reporting System; JADER, Japanese Adverse Drug Event Report.

In FAERS, more than 20% (535/2551) cases of ILD had fatal outcomes. T-Dxd had the highest mortality rate (30.89%, 114/369) (Fig. 4A). In JADER, T-Dxd not only had the highest mortality rate at 23.84% but also the highest number of fatal cases (36/151) (Fig. 4B). At the SMQ level, the majority of fatal cases were attributed to ILD and pneumonitis, accounting for 260 (23.55%) and 199 (20.49%), respectively (eFigure 3). Furthermore, our analysis in JADER revealed that the group with ILD had a significantly higher risk of fatality (8.82%, 134/1519) compared to the group without ILD (6.58%, 409/6219), *P* = 0.0030 (Fig. 4D). However, no analogous findings were observed in FAERS (ILD: 20.97%, 535/2551 vs. other PT: 22.05%, 16269/73783; *P* = 0.20) (Fig. 4C).

### Comparison with chemotherapy

**Fig. 5:**
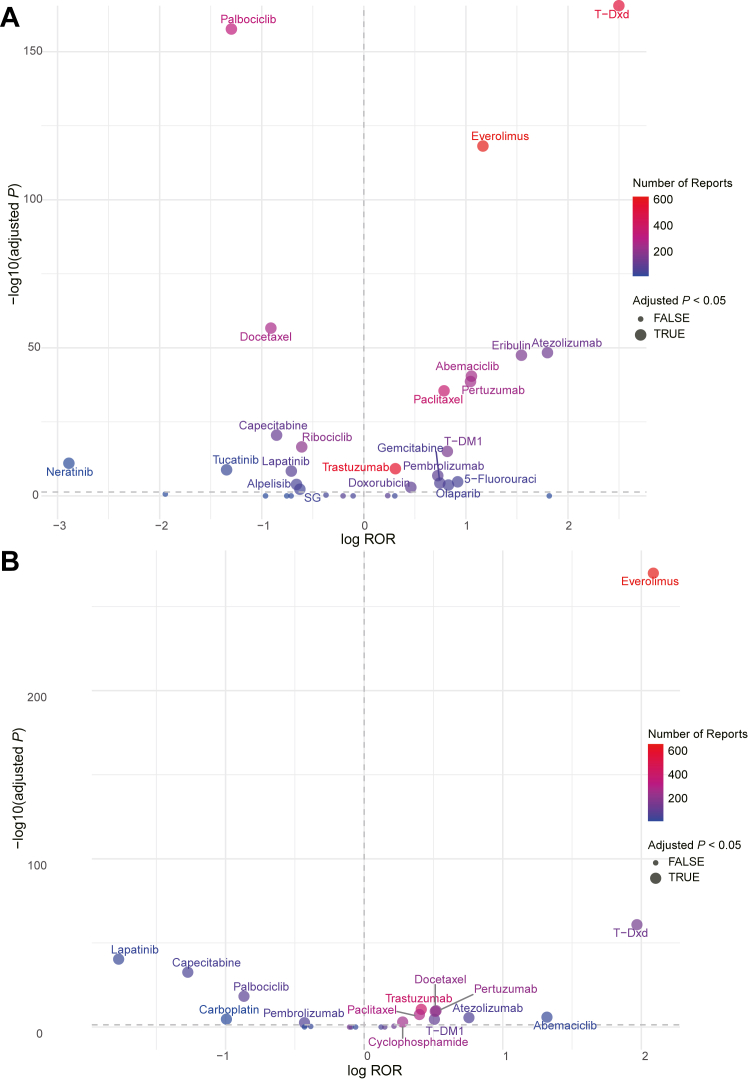
**Volcano plot comparing ILD signal between novel antineoplastic agents and chemotherapeutic drugs**. (A) and (B) show the comparison between novel antineoplastic agents and chemotherapeutic drugs in the FAERS and JADER databases, respectively. The x-axis represents the logarithm of the ROR, and the y-axis represents the negative base-10 logarithm of the adjusted *P* value, obtained from Fisher's exact test followed by Bonferroni correction. The colour intensity of each point indicates the number of reports, with warmer (redder) colours representing higher numbers of reports. Drugs in the upper right quadrant exhibit both higher ILD signals and significant statistical differences. Abbreviations: T-DM1, trastuzumab emtansine; T-Dxd, trastuzumab deruxtecan; Chisq, chi-square; ROR, reporting odds ratio; ILD, interstitial lung disease; FAERS, FDA Adverse Event Reporting System; JADER, Japanese Adverse Drug Event Report.

Overall, there are more signals of disproportionate reporting for novel antineoplastic agents compared with conventional chemotherapeutic agents (Fig. 5), details in Appendix (eFigures 4 and 5).

### Factors associated with ILD

Multivariate Cox regression analysis showed that increasing age raises the odds of reporting for ILD, while increasing weight lowers the odds (eTable 7), additionally, the number of drugs taken showed no significant impact on ILD. Details in Appendix.

### Disproportionality analysis reported by medical professionals

To enhance data quality and reduce erroneous reports, we excluded cases reported by non-professionals such as consumers and lawyers. Disproportionality analysis was specifically conducted on ILD cases reported by healthcare professionals, including physicians, pharmacists, and nurses. The analysis demonstrated that, in both databases, the drugs signal of disproportionate reporting as reported by healthcare professionals are generally consistent with those identified in the overall reports by all personnel (eFigures 6 and 7).

## Discussion

This is currently the first large sample real-world study on ILD associated with novel antineoplastic agents, encompassing the widest range of drugs and the largest volume of data. Our results showed that mAbs, ADCs, PD-1/PD-L1 inhibitors, and mTOR inhibitors were associated with ILD, although not found in PARP inhibitors and CDK4/6 inhibitors, there remains a risk of occurrence that should not be ignored.

Real-world studies, with larger sample sizes, diverse demographics, and longer durations, enhance generalizability and provide a more accurate reflection of adverse event frequency and nature in clinical practice. Our study analysed both the U.S. and Japanese adverse reaction databases, providing a richer dataset, and a more diverse population and ethnicity, which improves the reliability of results. Currently, several researches have demonstrated that various drugs are associated with a higher incidence of ILD.^8^^,^^12^ Additionally, various real-world studies, through analyzing hospital case and adverse reaction databases, have evaluated ILD associated with antineoplastic agents.^32^ However, no study has yet analysed the association between ILD and all novel antineoplastic drugs, particularly in patients with breast cancer, who represent one of the primary groups treated with these agents. Moreover, existing studies typically focus on ILDs caused by individual drugs and fail to analyse the signals and outcomes of ILDs caused by different classes of antineoplastic drugs.^26^

Some novel antineoplastic drugs are strongly associated with ILD development while others rarely induce ILD. Both disproportionality analysis and Cox analysis showed this is closely related to the type of drug. Specifically, in both databases, most of the drugs with signal of disproportionate reporting were concentrated in the classes of mAbs, ADCs, PD-1/PD-L1 inhibitors, and mTOR inhibitors. As an ADC, T-Dxd showed the consistent signal of ILD in both databases, in line with previous clinical studies. Indeed, a significant number of ILD cases linked to T-Dxd were reported during the DESTINY clinical trials, and our previous meta-analysis also identified a notable incidence of pneumonitis/ILD with ADCs.^13^^,^^19^ Concurrently, FDA issued a black box warning, its most stringent alert, to identify ILD as a potential severe adverse reaction to T-Dxd, and requires particular attention.^33^ Like T-Dxd, T-DM1 also exhibited similar signal for ILD and targets the human epidermal growth factor receptor 2 (HER2) protein. Given that the target of ILD is at the level of the alveolar epithelium or capillary endothelium, this targeting could explain the occurrence of ILD associated with these drugs. However, its signal value was different from that of T-Dxd, which can be attributed to its distinct payload (DM1 vs. deruxtecan), more stable linker (uncleavable vs. cleavable), and lower drug-antibody ratio (3.5:1 vs. 8:1). Unlike T-Dxd and T-DM1, sacituzumab govitecan (SG) targets the trophoblast cell surface antigen-2 (TROP-2) receptor and uses a different payload (SN-38).^13^ This distinct may explain why SG didn't exhibit an ILD signal. Similarly, trastuzumab and pertuzumab are mAbs targeting the HER2 receptor. Despite the previously low reported incidence of ILD, pertuzumab exhibited ILD signals in our analysis, additionally, ILD has been included as a black box warning for trastuzumab.^34^^,^^35^ PD-1/PD-L1 inhibitors activate the immune system, which can lead to the attack on normal cells and tissues, may result in ILD. A meta-analysis reported the incidence of ILDs associated with PD-1 and PD-L1 inhibitors to be 3.6% and 1.3%, respectively.^36^ The mTOR inhibitor everolimus is recognized as one of the drugs with the highest incidence of drug-associated ILD (3%–58%), a finding that aligns with our results.^11^ It is noteworthy that among CDK4/6 inhibitors, typically not linked to ILD, abemaciclib is an exception, showing a signal of disproportionate reporting compared to palbociclib and ribociclib. A meta-analysis reported ILD incidence rates of 0.5% for palbociclib, 2.5% for abemaciclib, and 1.1% for ribociclib. The underlying mechanisms are not yet clear, but the ILD signal with abemaciclib may be due to its broader kinase inhibition and high lipophilicity, leading to greater accumulation in lung tissues, and its continuous dosing regimen, which could raise drug concentrations and the likelihood of adverse effects.^14^

Through sensitivity analysis, we found that both in the SMQ analysis of ILD and in the analysis using ILD as the sole PT for case selection, disproportionate reporting signals for the five novel antineoplastic agents—T-Dxd, abemaciclib, everolimus, T-DM1, and pertuzumab—were consistently identified in both the FAERS and JADER databases. Moreover, the results were highly consistent before and after adjustment. This cross-database and cross-PT consistency further reinforces the reliability of the potential association between these drugs and ILD. These results highlight the need for increased vigilance regarding the occurrence of ILD during the clinical use of these drugs, along with close monitoring to ensure timely recognition and management. In addition, the sensitivity analysis of de-duplicated cases in JADER revealed that all drugs, except for pertuzumab, were consistent with the findings from the above analysis.

In both databases, the TTO for ILD was different for the various drugs. However, over half of the ILD cases occurred within the first three months of treatment, indicating most ILD events arise early, consistent with previous studies.^11^ Moreover, the median onset time for fatal cases was significantly shorter than that for all cases, indicating a more rapid progression in fatal cases. The reason for this outcome may be that these cases are inherently more severe and the patients have poorer underlying health conditions, such as pre-existing lung damage, which may make them more susceptible to drug side effects, thus accelerating the course of the disease, this further illustrates the importance of early recognition interventions for drug-induced ILD. The WSP test revealed that the occurrence of ILD for most drugs is not time-dependent, indicating that ILD could occur at any stage of treatment. However, in FAERS, mAbs such as trastuzumab and pertuzumab, along with CDK4/6 inhibitors like palbociclib, ribociclib, and abemaciclib, the odds of ILD was lower than in the early post-dose period. The reason for this pattern may relate to the different mechanisms of action and biological activities of these drugs, necessitating further studies to elucidate. Therefore, while it is crucial to pay extra attention to ILD in the early stages of treatment, continuous monitoring throughout treatment is essential to ensure that no potential cases are missed at any stage.

Analyzing fatal cases revealed that, with the exception of SG, ADCs and ICIs had relatively high mortality rates in cases of developing ILD, consistent with previous studies.^19^^,^^37^ Unexpectedly, mAbs and CDK4/6 inhibitors, which have been rarely reported or not reported in previous studies related to ILD deaths, demonstrated non-negligible mortality.^14^^,^^38^ Although not all deaths are necessarily due to ILD, in the real-world setting, where patients often have complex health conditions and treatment histories, these risks became apparent. Moreover, the SMQ for ILD was applied in this study, enhancing the case identification rate and sensitivity by encompassing a broad spectrum of clinically related terms. This also accounts for the higher mortality rates observed in databases compared to previous studies. Notably, in FAERS, the ILD mortality rate of everolimus was significantly higher than in JADER. This variation could stem from differences in reporting behaviours, patient health baselines, and medical interventions, highlighting the importance of accounting for regional differences in global drug safety monitoring. In JADER, patients with ILD had a significantly higher mortality rate compared to those without ILD. This result suggests that ILD may be a key factor in causing death and emphasizes the importance of monitoring ILD as an early indicator of severe outcomes during treatment. It is important to acknowledge that the causal relationship between reported fatal cases and ILD cannot be definitively established. Many deaths reported in both databases may be influenced by other factors, such as comorbidities, disease progression, or the underlying health conditions of patients with cancer. Furthermore, selective reporting of severe cases, coupled with variations in reporting practices across databases, complicates the interpretation of mortality data. Nevertheless, analyzing mortality reports offers a useful reference for understanding real-world situation of ILD. These findings highlight the need for further monitoring and evaluation of ILD in patients receiving novel antineoplastic agents.

Novel antineoplastic agents are more likely to be reported ILD than conventional chemotherapeutic agents. In addition, age and weight have a significant effect on the incidence of ILD (Appendix).^39^

A strengthening of the study's reliability was achieved by analyzing specific ILD reports. Even when considering only reports from medical professionals, the drugs signals of disproportionate reporting were generally consistent with those identified in overall reports.

This study has several limitations.^40^ Firstly, although we adjusted for confounding factors such as age, weight, reporter type, and concomitant drug use, inherent reporting selectivity and incomplete information in the databases may still introduce biases. Importantly, the risk of coprescription bias cannot be fully addressed due to the complexity of drug combinations and incomplete data on concomitant drugs. Secondly, due to the lack of crucial clinical information in the databases, such as tumour stage, metastasis status, underlying lung diseases, and smoking history, residual unmeasured confounders may affect the accuracy of our results. Additionally, data derived from spontaneous reporting systems are suitable only for signal detection and correlation analysis and are insufficient for causal inference, with the causal relationship between reported fatal cases and ILD remaining unclear. Thirdly, missing drug dose data, non-uniformity of units, and the diversity of treatment regimens hinder the assessment of the dose-ILD relationship. Fourthly, since the primaryid numbers in FAERS and JADER are not universal, and due to differences in reporting formats, content, and languages, deduplication can only rely on limited information (e.g., gender, age, drug, event date, country), potentially leading to missed or incorrectly excluded cases. Finally, pharmacovigilance studies inherently face limitations, including underreporting, differences in reporting enthusiasm (e.g., enhanced monitoring after new drug launches), and insufficient coverage of late-onset adverse events, which may distort disproportionate signals (over- or underestimation) and hinder the detection of delayed adverse events. Taken together, these factors suggest that the findings of this study should be further validated through prospective cohort studies or real-world data to more comprehensively assess the ILD risk and incidence associated with novel antineoplastic agents.

In conclusion, multiple novel antineoplastic agents for breast cancer, specifically T-DXd, abemaciclib, everolimus, T-DM1, and pertuzumab, demonstrated signals of disproportionate reporting associated with ILD. The onset time for most drugs appeared early after administration, with fatal cases occurring even earlier. Novel antineoplastic drugs were more strongly associated with ILD than conventional chemotherapeutic agents. Age and weight were potential factors influencing the occurrence of ILD. These findings can help clinicians promptly identify and manage ILD, thereby improving patients' treatment persistence and quality of life.

## Contributors

ZZ, YL and JZ conceived and designed the study. CZ, QD, ZL, DR, FZ and JZ provided study materials. ZZ and YZ collected the data. ZZ, LZ and DR conducted statistics on the data. ZZ, CZ, QD and JZ conducted statistics on the data. ZZ and JZ drafted the manuscript. YZ and FZ directly accessed and verified the data in the manuscript. All authors had full access to the data in the study. JZ had final responsibility for the decision to submit for publication.

## Data sharing statement

This study is based on the FAERS and JADER databases, which are publicly accessible at https://fis.fda.gov/extensions/FPD-QDE-FAERS/FPD-QDE-FAERS.html and https://www.info.pmda.go.jp/fukusayoudb/CsvDownload.jsp without requiring prior applications. Further information is available from the corresponding author upon request.

## Declaration of interests

All authors have declared no conflicts of interest.

## Appendix A Supplementary data

Supplementary

Tracked changes Supplementary
