# Supplementary material for: Disproportionality analysis of interstitial lung disease associated with novel antineoplastic agents during breast cancer treatment: a pharmacovigilance study
Source: eClinicalMedicine. 2025 Mar 18;82:103160. doi: 10.1016/j.eclinm.2025.103160 (PMC11957809; doi:10.1016/j.eclinm.2025.103160)
Supplement: Supplementary [file mmc1.pdf]

Supplementary

Additions to the manuscript

Methods

Statistical analysis

The following formula was used to calculate the ROR and 95% confidence interval (CI):

$$ROR = \frac{a/c}{b/d}$$

$$95\%\ CI = e^{\ln(ROR) \pm 1.96\sqrt{\frac{1}{a}+\frac{1}{b}+\frac{1}{c}+\frac{1}{d}}}$$

A represents reports of target events involving target drugs; b represents reports of non-target events involving target drugs; c represents reports of target events involving drugs other than the target drugs; d represents reports of non-target events involving drugs other than the target drugs.

Results

Disproportionality analysis

In FAERS, we further conducted a signal detection analysis for the top 10 most frequently reported PTs under the SMQ level (Figure 2C). PTs such as interstitial lung disease (ROR 1.23; 95% CI 1.13-1.33), pneumonitis (ROR 1.75; 95% CI 1.60-1.91), pulmonary fibrosis (ROR 1.49; 95% CI 1.21-1.85), pulmonary toxicity (ROR 1.50; 95% CI 1.16-1.95), lung opacity (ROR 3.02; 95% CI 1.98-4.16), and immune-mediated lung disease (ROR 2.78; 95% CI 1.07-7.24) all showed signal of disproportionate reporting of novel antineoplastic agents. Among these, interstitial lung disease (n=1,214) and pneumonitis (n=1,183) accounted for the vast majority of the reports. In the JADER database, we performed disproportionality and sensitivity analyses

after excluding cases suspected of being duplicates of FAERS database. A total of 4 novel antineoplastic agents showed a signal of disproportionate reporting for ILD both before and after adjustment: Everolimus [ROR 6.57 (5.88-7.34), aROR 2.49 (1.62-3.84)]; T-Dxd [ROR 4.59 (3.61-5.79), aROR 11.05 (8.07-15.13)]; abemaciclib [ROR 2.37 (1.36-3.90), aROR 2.34 (1.11-4.91)]; T-DM1 [ROR 1.53 (1.24-1.88), aROR 1.62 (1.13-2.33)] (eTable 2).

Comparison with chemotherapy

In FAERS, 5 chemotherapeutic drugs were identified as signal of disproportionate reporting for ILD: Eribulin (ROR 4.68; 95% CI 3.97-5.53), 5-fluorouracil (ROR 2.51; 95% CI 1.81-3.47), paclitaxel (ROR 2.19; 95% CI 1.97-2.45), gemcitabine (ROR 2.11; 95% CI 1.59-2.80) and doxorubicin (ROR 1.59; 95% CI 1.28-1.97) (eFigure 4). In JADER, 4 drugs were confirmed: Docetaxel (ROR 1.67; 95% CI 1.45-1.93), paclitaxel (ROR 1.49; 95% CI 1.31-1.69), cyclophosphamide (ROR 1.32; 95% CI 1.15-1.51), and eribulin (ROR 1.24; 95% CI 1.03-1.48) (eFigure 5). It is evident that, from an overall perspective, there are more signals of disproportionate reporting for novel antineoplastic agents compared with conventional chemotherapeutic agents, as shown in the volcano plots from both the FAERS (Figure 5A) and JADER (Figure 5B). Furthermore, in FAERS, univariate logistic regression analysis showed that novel antineoplastic agents were significantly associated with the occurrence of ILD compared to conventional chemotherapeutic agents (OR = 2.47, 95% CI: 2.16-2.81, $P < 0.0001$). Similarly, in JADER, novel antineoplastic agents were also significantly associated with ILD compared to conventional chemotherapeutic agents (OR = 1.61, 95% CI: 1.37-1.88, $P < 0.0001$). These findings suggest stronger reporting associations between novel antineoplastic agents and ILD than those observed with conventional chemotherapeutic agents.

Factors associated with ILD

Results of multivariate Cox regression analysis from FAERS indicated that both age and weight were potential factors influencing the occurrence of ILD. Specifically, each additional year was associated with an increase in the odds of reporting ILD by approximately 0.97% (HR = 1.0097, 95% CI: 1.0036-1.0159, $P = 0.0020$), while each additional kilogram of body weight was associated with a decrease in the odds of reporting ILD by approximately 2.17% (HR = 0.9783, 95% CI: 0.9729-0.9836, $P < 0.0001$). In contrast, the number of drugs taken showed no significant impact on ILD (HR = 1.0036, 95% CI: 0.9918-1.0155, $P = 0.55$). The analysis from the JADER database also confirmed the consistency of age as a potential factor, with each additional year increasing the odds of reporting ILD by about 1.83% (HR = 1.0183, 95% CI: 1.0094-1.0270, $P < 0.0001$). However, neither weight (HR = 1.0077, 95% CI: 0.9990-1.0170, $P = 0.081$) nor the number of drugs taken (HR = 0.9924, 95% CI: 0.9757-1.0090, $P = 0.038$) showed statistically significant associations with ILD (eTable 7).

Disproportionality analysis reported by medical professionals

In the FAERS and JADER databases, reports are predominantly submitted by healthcare professionals, pharmaceutical companies, and consumers. To enhance data quality and reduce erroneous reports, we excluded cases reported by non-professionals such as consumers and lawyers. Disproportionality analysis was specifically conducted on ILD cases reported by healthcare professionals, including physicians, pharmacists, and nurses. The analysis demonstrated that, regardless of whether it is from the FAERS or JADER database, the drugs associated with ILD as reported by healthcare professionals are generally consistent with those identified in the overall reports by all personnel. (eFigure 6 and eFigure 7).

Discussion

We found that novel antineoplastic agents are more strongly associated with ILD compared to traditional chemotherapy drugs, emphasizing the need to be vigilant

about potential pulmonary toxicity when using these new drugs. This finding also reminds us that while the efficacy of new drugs is important, it is equally crucial to thoroughly assess and monitor their potential serious adverse effects.

In analyzing potential factors for ILD, both age and weight significantly impact the incidence of ILD. With increasing age, due to the typically weaker immune function and chronic inflammatory states in elderly populations, the odds of ILD also increases. Conversely, an increase in weight is negatively correlated with the odds of ILD. This observation aligns with existing studies where a higher BMI and body weight have been linked to lower mortality rates and slower disease progression, suggesting better nutritional status and greater energy reserves that aid in managing chronic illnesses.[30] Additionally, higher body weight might be associated with greater lung capacity and enhanced respiratory functions, offering certain protective benefits. The number of concomitant drugs did not significantly impact ILD, likely due to the inclusion of supportive or pre-treatment drugs, which are less likely to induce ILD. Additionally, only ADR reports categorized as “primary suspect” or “suspected medicine” were analysed, focusing on drugs most likely responsible for ILD. This may have reduced the influence of the total drug count on ILD. These findings underscore the necessity of incorporating age and weight considerations into therapeutic strategies for ILD patients to optimize outcomes.

References

40. Comes A, Wong AW, Fisher JH, et al: Association of BMI and Change in Weight with Mortality in Patients with Fibrotic Interstitial Lung Disease. Chest 161:1320–1329, 2022

Figure legends

eFigure 1. Cox regression analysis of ILD reports across different classes of novel antineoplastic agents in the FAERS database.

Abbreviations: HR, hazard ratio; TKIs, tyrosine kinase inhibitors; mAbs, monoclonal

antibodies; ADCs, antibody-drug conjugates; PD-1/PD-L1, programmed death-1/programmed death-ligand 1; CDK4/6, cyclin-dependent kinase 4/6; PARP, poly ADP-ribose polymerase; PI3K/AKT/mTOR, phosphatidylinositol 3-kinase/protein kinase B/mammalian target of rapamycin.

eFigure 2. Kaplan-Meier analysis of the probability of developing ILD between the drug groups and the control group. TKIs (A), mAbs (B), ADCs (C), ICIs (D), CDK4/6 inhibitors (E), PARP inhibitors (F), PI3K/AKT/mTOR inhibitors (G). Each curve compares the probability of ILD occurrence over time for patients treated with a specific drug class against other drugs.

Abbreviations: HR, hazard ratio; TKIs, tyrosine kinase inhibitors; mAbs, monoclonal antibodies; ADCs, antibody-drug conjugates; PD-1/PD-L1, programmed death-1/programmed death-ligand 1; CDK4/6, cyclin-dependent kinase 4/6; PARP, poly ADP-ribose polymerase; PI3K/AKT/mTOR, phosphatidylinositol 3-kinase/protein kinase B/mammalian target of rapamycin.

eFigure 3. A column plot showing the fatality proportions and number for the different SMQs in the FAERS database.

Abbreviations: SMQ, standardized MedDRA query; FAERS, FDA Adverse Event Reporting System.

eFigure 4. Forest plot of ROR values for different chemotherapeutic agents associated with ILD in the FAERS database.

Abbreviations: ROR, reporting odds ratio; CI, confidence interval; ILD, interstitial lung disease; FAERS, FDA Adverse Event Reporting System.

eFigure 5. Forest plot of ROR values for different chemotherapeutic agents associated with ILD in the JADER database.

Abbreviations: ROR, reporting odds ratio; CI, confidence interval; ILD, interstitial lung disease; JADER, Japanese Adverse Drug Event Report.

eFigure 6. Forest plot of ROR values for different novel antineoplastic agents associated with ILD in the FAERS database, based on reports from healthcare professionals.

Abbreviations: ROR, reporting odds ratio; CI, confidence interval; ILD, interstitial lung disease; FAERS, FDA Adverse Event Reporting System.

eFigure 7. Forest plot of ROR values for different novel antineoplastic agents associated with ILD in the JADER database, based on reports from healthcare professionals.

Abbreviations: ROR, reporting odds ratio; CI, confidence interval; ILD, interstitial lung disease; JADER, Japanese Adverse Drug Event Report.

eTable 1. The PTs included in the narrow SMQ of ILD

Abbreviations: PT, preferred term; SMQ, standardized MedDRA query; ILD, interstitial lung disease.

eTable 2. ROR and aROR for ILD associated with antineoplastic agents in breast cancer treatment using the JADER database after exclusion of suspected duplicate cases.

Abbreviations: ROR, reporting odds ratio; aROR, adjusted reporting odds ratio; ILD, interstitial lung disease; JADER, Japanese Adverse Drug Event Report; CI, confidence interval.

eTable 3. Raw data used in the disproportionality analysis to calculate the ROR

Abbreviations: ROR, reporting odds ratio; a, reports of target events involving target drugs; b, reports of non-target events involving target drugs; c, reports of target events involving drugs other than the target drugs; d, reports of non-target events involving drugs other than the target drugs; FAERS, FDA Adverse Event Reporting System; TKIs, tyrosine kinase inhibitors; mAbs, monoclonal antibodies; ADCs, antibody-drug

conjugates; PD-1/PD-L1, programmed death-1/programmed death-ligand 1; CDK4/6, cyclin-dependent kinase 4/6; PARP, poly ADP-ribose polymerase; PI3K/AKT/mTOR, phosphatidylinositol 3-kinase/protein kinase B/mammalian target of rapamycin; JADER, Japanese Adverse Drug Event Report; SMQ, standardized MedDRA query.

eTable 4. Top 50 concomitant drugs with novel antineoplastic agents in the FAERS database.

Abbreviations: FAERS, FDA Adverse Event Reporting System.

eTable 5. ROR and aROR for novel antineoplastic agents in breast cancer treatment using ILD as the sole PT.

Abbreviations: ROR, reporting odds ratio; aROR, adjusted reporting odds ratio; ILD, interstitial lung disease; PT, preferred term; CI, confidence interval; FAERS, FDA Adverse Event Reporting System; JADER, Japanese Adverse Drug Event Report.

eTable 6. Time to onset and Weibull shape parameters for interstitial lung disease associated with novel antineoplastic agents in FAERS and JADER databases.

Abbreviations: FAERS, FDA Adverse Event Reporting System; JADER, Japanese Adverse Drug Event Report; TTO, time to onset; IQR, interquartile range; CI, confidence interval; T-DM1, trastuzumab emtansine; T-Dxd, trastuzumab deruxtecan; SG, sacituzumab govitecan.

eTable 7. Cox regression analysis of clinical characteristics on ILD reports in FAERS and JADER.

Abbreviations: ILD, interstitial lung disease; FAERS, FDA Adverse Event Reporting System; JADER, Japanese Adverse Drug Event Report; HR, hazard ratio.

Drugs	HR (95% CI)	*P* value
TKIs	0.57 (0.42-0.76)	0.0001
mAbs	1.04 (0.90-1.20)	0.60
ADCs	2.83 (2.39-3.35)	< 0.0001
PD-1/PD-L1 inhibitors	2.17 (1.72-2.72)	< 0.0001
CDK4/6 inhibitors	0.34 (0.30-0.39)	< 0.0001
PARPis	1.42 (0.87-2.33)	0.16
PI3K/AKT/mTOR inhibitors	2.84 (2.46-3.28)	< 0.0001

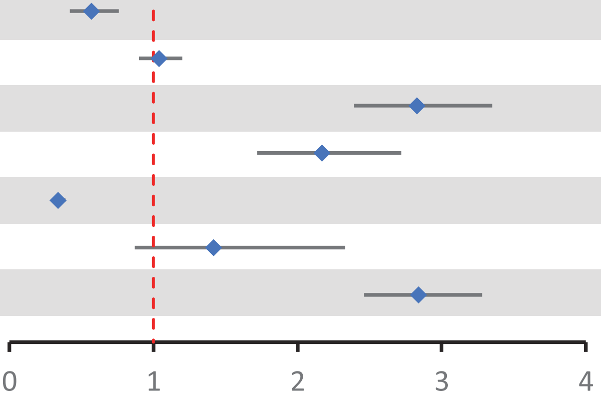

eFigure 1. Cox regression analysis of ILD reports across different classes of novel antineoplastic agents in the FAERS database.

Abbreviations: HR, hazard ratio; TKIs, tyrosine kinase inhibitors; mAbs, monoclonal antibodies; ADCs, antibody-drug conjugates; PD-1/PD-L1, programmed death-1/programmed death-ligand 1; CDK4/6, cyclin-dependent kinase 4/6; PARP, poly ADP-ribose polymerase; PI3K/AKT/mTOR, phosphatidylinositol 3-kinase/protein kinase B/mammalian target of rapamycin.

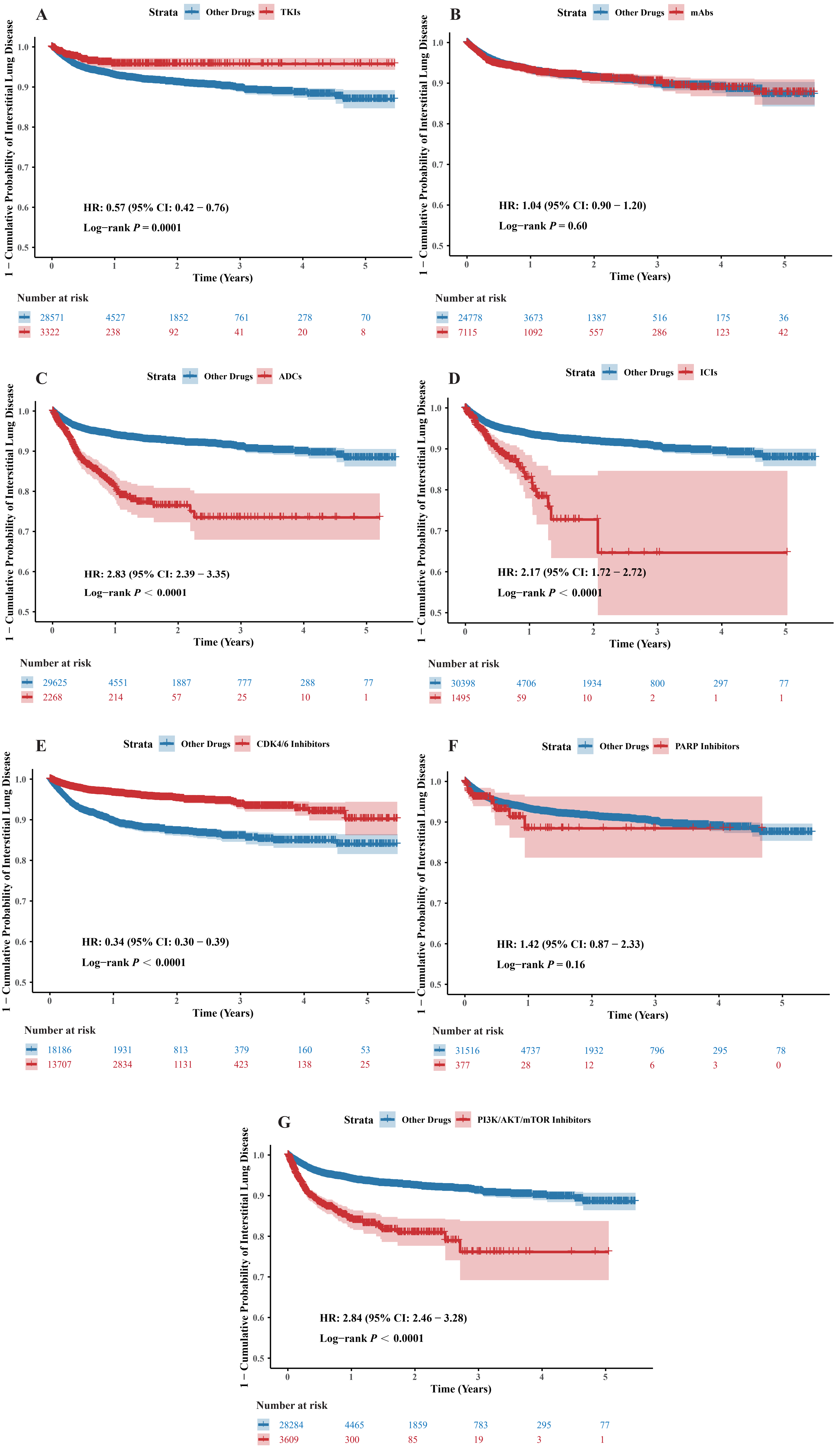

eFigure 2. Kaplan-Meier analysis of the probability of developing ILD between the drug groups and the control group. TKIs (A), mAbs (B),ADCs (C), ICIs (D), CDK4/6 inhibitors (E), PARP inhibitors (F), PI3K/AKT/mTOR inhibitors (G). Each curve compares the probability of ILD occurrence over time for patients treated with a specific drug class against other drugs.

Abbreviations: HR, hazard ratio; TKIs, tyrosine kinase inhibitors; mAbs, monoclonal antibodies; ADCs, antibody-drug conjugates; PD-1/PD-L1, programmed death-1/programmed death-ligand 1; CDK4/6, cyclin-dependent kinase 4/6; PARP, poly ADP-ribose polymerase; PI3K/AKT/mTOR, phosphatidylinositol 3-kinase/protein kinase B/mammalian target of rapamycin.

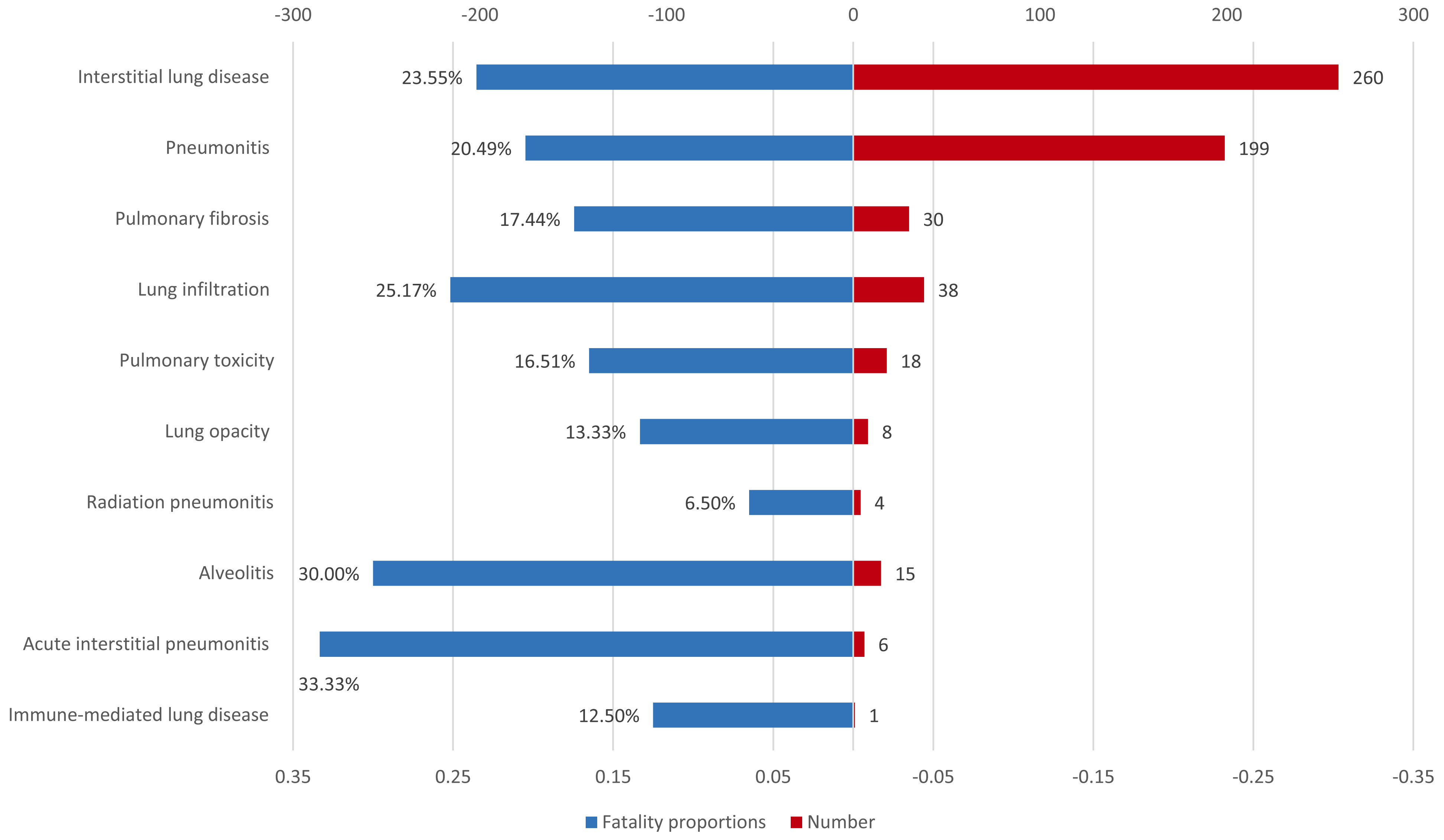

eFigure 3. A column plot showing the fatality proportions and number for the different SMQs in the FAERS database. Abbreviations: SMQ, standardized MedDRA query; FAERS, FDA Adverse Event Reporting System.

Drugs	Cases	ROR (95% CI)
Paclitaxel	352	2.19 (1.97-2.45)
Nab-paclitaxel	36	0.90 (0.65-1.25)
Docetaxel	247	0.40 (0.35-0.46)
Carboplatin	32	0.69 (0.49-0.98)
Cisplatin	1	0.38 (0.05-2.72)
Doxorubicin	83	1.59 (1.28-1.97)
Epirubicin	71	1.26 (1.00-1.60)
Capecitabine	100	0.43 (0.35-0.52)
Gemcitabine	49	2.11 (1.59-2.80)
Vinorelbine	21	1.35 (0.88-2.08)
Cyclophosphamide	65	0.82 (0.64-1.04)
Eribulin	147	4.68 (3.97-5.53)
5-Fluorouracil	37	2.51 (1.81-3.47)
Ixabepilone	5	0.49 (0.20-1.18)

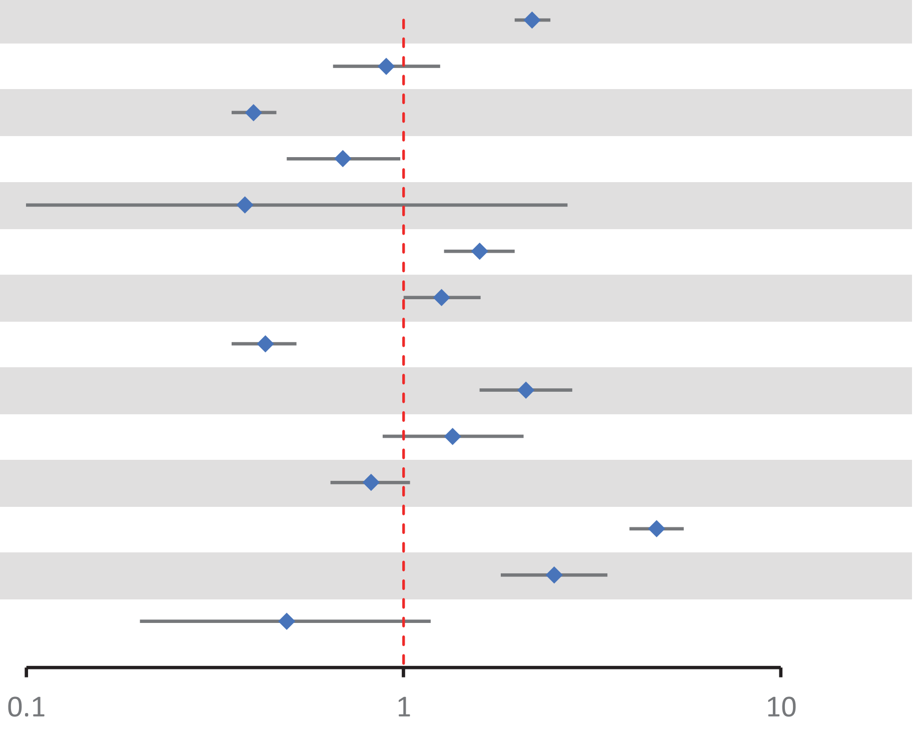

eFigure 4. Forest plot of ROR values for different chemotherapeutic agents associated with ILD in the FAERS database.
Abbreviations: ROR, reporting odds ratio; CI, confidence interval; ILD, interstitial lung disease; FAERS, FDA Adverse Event Reporting System.

Drugs	Cases	ROR (95% CI)
Paclitaxel	302	1.49 (1.31-1.69)
Nab-paclitaxel	61	0.91 (0.70-1.19)
Docetaxel	231	1.67 (1.45-1.93)
Carboplatin	18	0.37 (0.23-0.59)
Doxorubicin	57	1.13 (0.86-1.49)
Epirubicin	158	0.90 (0.76-1.06)
Capecitabine	64	0.28 (0.21-0.35)
Gemcitabine	25	0.94 (0.62-1.41)
Vinorelbine	30	0.68 (0.47-0.98)
Cyclophosphamide	261	1.32 (1.15-1.51)
Eribulin	133	1.24 (1.03-1.48)
5-Fluorouracil	100	1.16 (0.94-1.42)

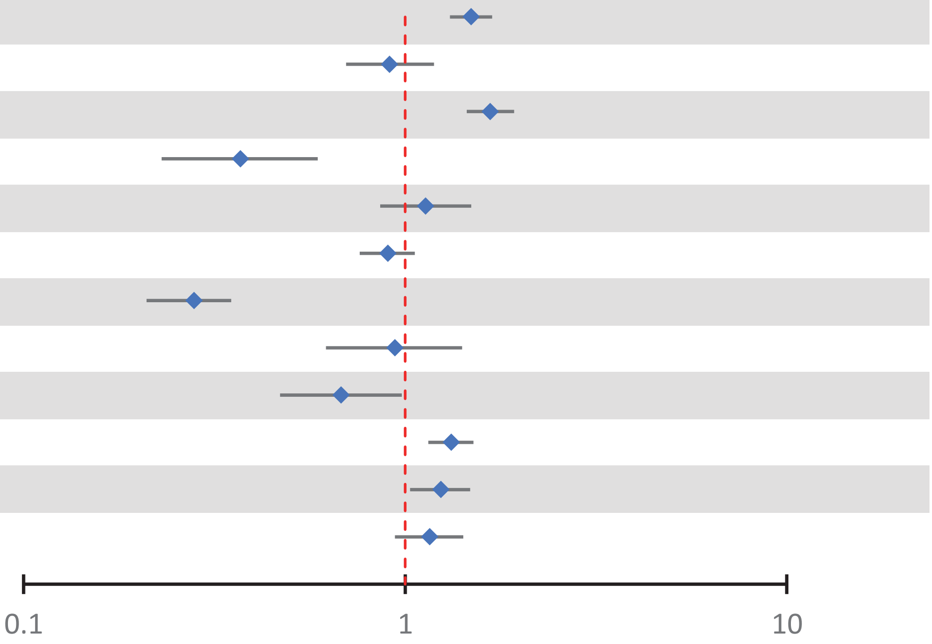

eFigure 5. Forest plot of ROR values for different chemotherapeutic agents associated with ILD in the JADER database.
Abbreviations: ROR, reporting odds ratio; CI, confidence interval; ILD, interstitial lung disease; JADER, Japanese Adverse Drug Event Report.

Drugs	Cases	ROR (95% CI)
**TKIs**	**59**	**0.31 (0.24-0.39)**
Lapatinib	49	0.53 (0.40-0.71)
Neratinib	2	0.04 (0.01-0.17)
Tucatinib	8	0.16 (0.08-0.32)
**mAbs**	**735**	**1.30 (1.20-1.41)**
Trastuzumab	512	1.06 (0.97-1.17)
Pertuzumab	223	2.27 (1.98-2.60)
**ADCs**	**639**	**3.61 (3.31-3.93)**
Trastuzumab emtansine	131	1.96 (1.64-2.34)
Trastuzumab deruxtecan	479	9.95 (8.98-11.03)
Sacituzumab govitecan	29	0.38 (0.27-0.55)
**PD-1/PD-L1 inhibitors**	**181**	**2.55 (2.19-2.96)**
Pembrolizumab	67	1.48 (1.16-1.89)
Atezolizumab	113	4.34 (3.59-5.25)
Avelumab	1	4.06 (0.55-29.89)
**CDK4/6 inhibitors**	**489**	**0.43 (0.40-0.48)**
Palbociclib	231	0.27 (0.24-0.31)
Ribociclib	132	0.54 (0.47-0.63)
Abemaciclib	126	2.59 (2.16-3.10)
**PARPis**	**30**	**1.83 (1.27-2.63)**
Olaparib	27	2.75 (1.87-4.03)
Talazoparib	2	0.47 (0.12-1.89)
Niraparib	1	0.46 (0.06-3.26)
**PI3K/AKT/mTOR inhibitors**	**526**	**2.43 (2.21-2.66)**
Alpelisib	29	0.57 (0.39-0.82)
Everolimus	497	2.95 (2.69-3.24)

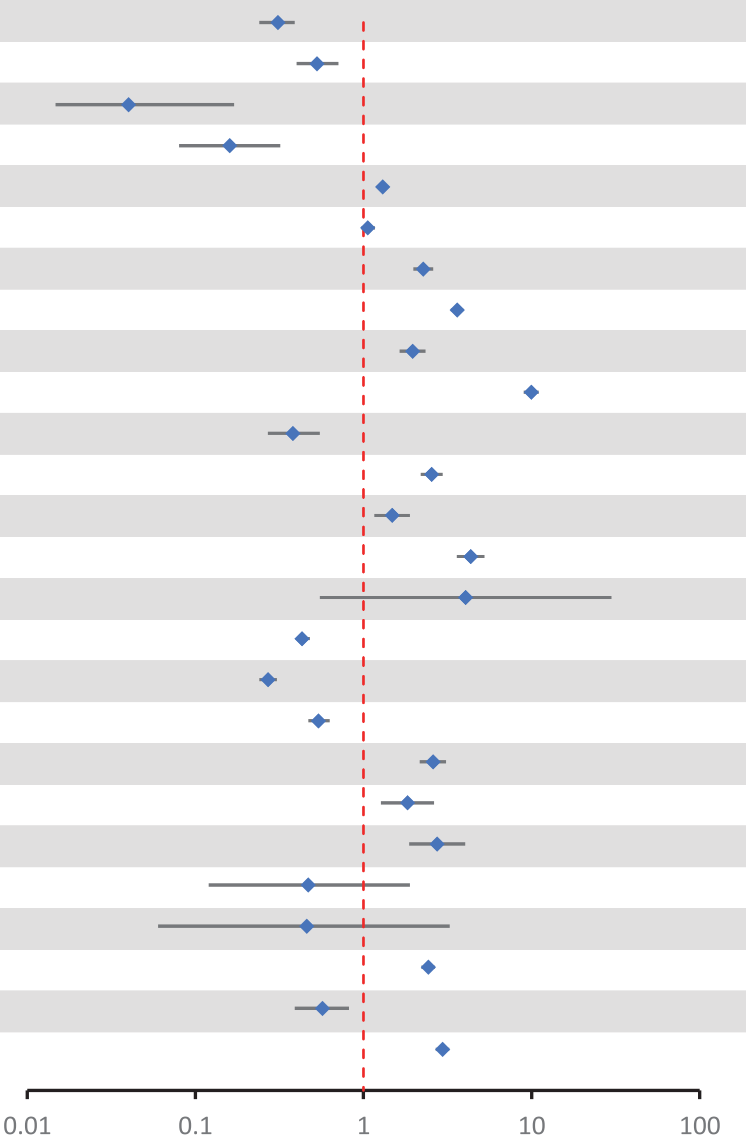

eFigure 6. Forest plot of ROR values for different novel antineoplastic agents associated with ILD in the FAERS database, based on reports from healthcare professionals.

Abbreviations: ROR, reporting odds ratio; CI, confidence interval; ILD, interstitial lung disease; FAERS, FDA Adverse Event Reporting System.

Drugs	Cases	ROR (95% CI)
Lapatinib	33	0.17 (0.12-0.24)
Trastuzumab	405	1.50 (1.34-1.68)
Pertuzumab	232	1.67 (1.45-1.93)
Trastuzumab emtansine	113	1.66 (1.35-2.03)
Trastuzumab deruxtecan	153	7.08 (5.77-8.68)
Pembrolizumab	71	0.63 (0.50-0.81)
Atezolizumab	67	2.13 (1.64-2.78)
Palbociclib	93	0.41 (0.33-0.51)
Abemaciclib	26	3.64 (2.34-5.67)
Olaparib	18	0.64 (0.40-1.04)
Everolimus	660	7.96 (7.16-8.85)

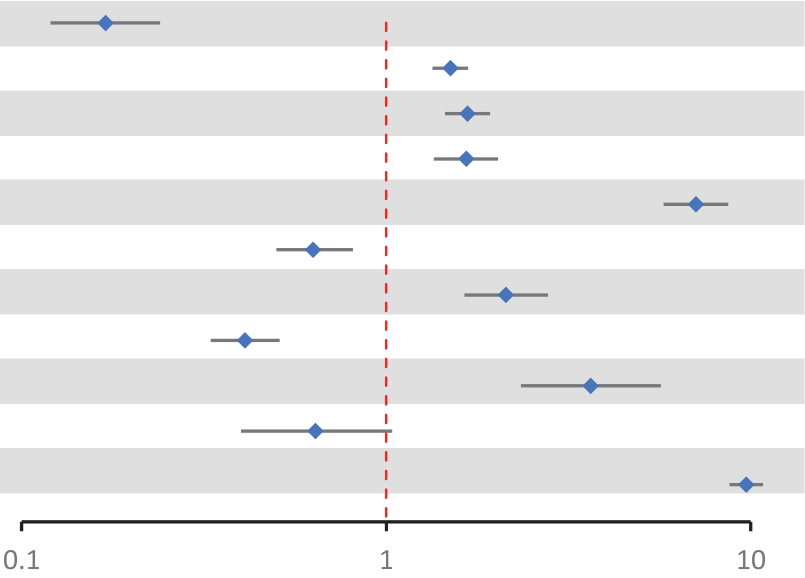

eFigure 7. Forest plot of ROR values for different novel antineoplastic agents associated with ILD in the JADER database, based on reports from healthcare professionals.

Abbreviations: ROR, reporting odds ratio; CI, confidence interval; ILD, interstitial lung disease; JADER, Japanese Adverse Drug Event Report.

eTable 1. The PTs included in the narrow SMQ of ILD

PT	code	grade	realm
Acute interstitial pneumonitis	10066728	PT	Narrow
Alveolar lung disease	10073344	PT	Narrow
Alveolar proteinosis	10001881	PT	Narrow
Alveolitis	10001889	PT	Narrow
Alveolitis necrotising	10050343	PT	Narrow
Autoimmune lung disease	10080701	PT	Narrow
Bronchiolitis	10006448	PT	Narrow
Bronchiolitis obliterans syndrome	10083303	PT	Narrow
Chronic graft versus host disease in lung	10086041	PT	Narrow
Combined pulmonary fibrosis and emphysema	10076515	PT	Narrow
Confirmed e-cigarette or vaping product use associated lung injury	10085189	PT	Narrow
Diffuse alveolar damage	10060902	PT	Narrow
Eosinophilia myalgia syndrome	10014952	PT	Narrow
Eosinophilic granulomatosis with polyangiitis	10078117	PT	Narrow
Eosinophilic pneumonia	10014962	PT	Narrow
Eosinophilic pneumonia acute	10052832	PT	Narrow
Eosinophilic pneumonia chronic	10052833	PT	Narrow
Hypersensitivity pneumonitis	10081988	PT	Narrow
Idiopathic interstitial pneumonia	10078268	PT	Narrow
Idiopathic pneumonia syndrome	10063725	PT	Narrow
Idiopathic pulmonary fibrosis	10021240	PT	Narrow
Immune-mediated lung disease	10085352	PT	Narrow
Interstitial lung abnormality	10087834	PT	Narrow
Interstitial lung disease	10022611	PT	Narrow
Low lung compliance	10086117	PT	Narrow
Lung infiltration	10025102	PT	Narrow
Lung opacity	10081792	PT	Narrow
Necrotising bronchiolitis	10070831	PT	Narrow
Obliterative bronchiolitis	10029888	PT	Narrow
Pleuroparenchymal fibroelastosis	10084305	PT	Narrow
Pneumonitis	10035742	PT	Narrow
Probable e-cigarette or vaping product use associated lung injury	10085188	PT	Narrow
Progressive massive fibrosis	10036805	PT	Narrow
Pulmonary fibrosis	10037383	PT	Narrow
Pulmonary necrosis	10058824	PT	Narrow
Pulmonary radiation injury	10061473	PT	Narrow
Pulmonary toxicity	10061924	PT	Narrow
Pulmonary vasculitis	10037457	PT	Narrow
Radiation alveolitis	10037754	PT	Narrow
Radiation bronchitis	10085628	PT	Narrow

Radiation fibrosis - lung	10037758	PT	Narrow
Radiation pneumonitis	10037765	PT	Narrow
Rheumatoid arthritis-associated interstitial lung disease	10085517	PT	Narrow
Small airways disease	10080547	PT	Narrow
Transfusion-related acute lung injury	10052235	PT	Narrow

Abbreviations: PT, preferred term; SMQ, standardized MedDRA query; ILD, interstitial lung disease.

eTable 2. ROR and aROR for ILD associated with antineoplastic agents in breast cancer treatment using the JADER database after exclusion of suspected duplicate cases.

Drugs	Cases	ROR (95% CI)	aROR (95% CI)	*P* value
Lapatinib	28	0.14 (0.10-0.20)	0.31 (0.18-0.53)	< 0.0001
Trastuzumab	318	1.17 (1.03-1.32)	1.19 (0.90-1.57)	0.23
Pertuzumab	200	1.42 (1.22-1.65)	1.05 (0.68-1.63)	0.82
Trastuzumab emtansine	105	1.53 (1.24-1.88)	1.62 (1.13-2.33)	0.009
Trastuzumab deruxtecan	98	4.59 (3.61-5.79)	11.05 (8.07-15.13)	< 0.0001
Pembrolizumab	62	0.56 (0.43-0.72)	1.49 (0.65-3.37)	0.34
Atezolizumab	55	1.75 (1.30-2.32)	1.48 (0.84-2.60)	0.17
Palbociclib	60	0.27 (0.20-0.34)	0.66 (0.42-1.04)	0.074
Abemaciclib	17	2.37 (1.36-3.90)	2.34 (1.11-4.91)	0.025
Olaparib	16	0.58 (0.34-0.93)	0.70 (0.28-1.76)	0.45
Everolimus	543	6.57 (5.88-7.34)	2.49 (1.62-3.84)	< 0.0001

Abbreviations: ROR, reporting odds ratio; aROR, adjusted reporting odds ratio; ILD, interstitial lung disease; JADER, Japanese Adverse Drug Event Report; CI, confidence interval.

eTable 3. Raw data used in the disproportionality analysis to calculate the ROR

Drug	a	b	c	d	ROR (95% CI)
FAERS					
**TKIs**					
Lapatinib	65	20845	5688	896610	0.49 (0.38-0.63)
Neratinib	2	6092	3764	637684	0.05 (0.01-0.22)
Tucatinib	15	8687	2675	403944	0.26 (0.16-0.43)
**mAbs**					
Trastuzumab	550	65085	5464	881123	1.36 (1.25-1.49)
Pertuzumab	231	13251	5055	824733	2.84 (2.49-3.25)
**ADCs**					
Trastuzumab emtansine	139	9863	5032	809336	2.27 (1.91-2.69)
Trastuzumab deruxtecan	516	7892	2437	453529	12.17 (11.04-13.41)
Sacituzumab govitecan	29	8244	2661	404387	0.53 (0.37-0.77)
**PD-1/PD-L1 inhibitors**					
Pembrolizumab	80	6347	4659	762791	2.06 (1.65-2.58)
Atezolizumab	118	3338	4090	699350	6.04 (5.02-7.28)
Avelumab	1	28	3900	671803	6.15 (0.84-45.23)
**CDK4/6 inhibitors**					
Palbociclib	319	161511	4285	592850	0.27 (0.24-0.31)
Ribociclib	184	56004	3717	615827	0.54 (0.47-0.63)
Abemaciclib	241	14998	3436	613882	2.87 (2.52-3.27)
**PARPis**					
Olaparib	36	2567	4643	758305	2.29 (1.65-3.19)
Talazoparib	2	706	3314	550023	0.47 (0.12-1.88)
Niraparib	1	1202	3900	670629	0.14 (0.02-1.02)
**PI3K/AKT/mTOR inhibitors**					
Alpelisib	39	12068	3113	498351	0.52 (0.38-0.71)
Everolimus	607	32426	5005	858763	3.21 (2.95-3.50)
JADER					
Lapatinib	34	2147	2541	27588	0.17 (0.12-0.24)
Trastuzumab	412	3329	2163	26406	1.51 (1.35-1.69)
Pertuzumab	236	1687	2339	28048	1.68 (1.45-1.93)
Trastuzumab emtansine	114	806	2461	28929	1.66 (1.36-2.03)
Trastuzumab deruxtecan	153	260	2422	29475	7.16 (5.84-8.78)
Pembrolizumab	72	1265	2503	28470	0.65 (0.51-0.82)
Atezolizumab	67	368	2508	29367	2.13 (1.64-2.77)
Palbociclib	95	2485	2480	27250	0.42 (0.34-0.52)
Abemaciclib	27	84	2548	29651	3.74 (2.42-5.78)
Olaparib	18	318	2557	29417	0.65 (0.40-1.05)
Everolimus	666	1234	1909	28501	8.06 (7.25-8.95)
SMQ					
Interstitial lung disease	1214	433276	1177	516555	1.23 (1.13-1.33)
Pneumonitis	1183	433307	807	516925	1.75 (1.60-1.91)

Drug	a	b	c	d	ROR (95% CI)
Pulmonary fibrosis	188	434302	150	517582	1.49 (1.21-1.85)
Lung infiltration	162	434328	218	517514	0.89 (0.72-1.09)
Pulmonary toxicity	130	434360	103	517629	1.50 (1.16-1.95)
Lung opacity	76	434414	30	517702	3.02 (1.98-4.61)
Radiation pneumonitis	66	434424	87	517645	0.90 (0.66-1.24)
Alveolitis	50	434440	93	517639	0.64 (0.45-0.90)
Acute interstitial pneumonitis	19	434471	33	517699	0.69 (0.39-1.21)
Immune-mediated lung disease	14	434476	6	517726	2.78 (1.07-7.24)

Abbreviations: ROR, reporting odds ratio; a, reports of target events involving target drugs; b, reports of non-target events involving target drugs; c, reports of target events involving drugs other than the target drugs; d, reports of non-target events involving drugs other than the target drugs; FAERS, FDA Adverse Event Reporting System; TKIs, tyrosine kinase inhibitors; mAbs, monoclonal antibodies; ADCs, antibody-drug conjugates; PD-1/PD-L1, programmed death-1/programmed death-ligand 1; CDK4/6, cyclin-dependent kinase 4/6; PARP, poly ADP-ribose polymerase; PI3K/AKT/mTOR, phosphatidylinositol 3-kinase/protein kinase B/mammalian target of rapamycin; JADER, Japanese Adverse Drug Event Report; SMQ, standardized MedDRA query.

eTable 4. Top 50 concomitant drugs with novel antitumor agents in the FAERS database

Drug	N	FDA Label information
Letrozole/Femara	15099	Not common
Fulvestrant/Faslodex	8738	Not common
Docetaxel/Taxotere	7515	**Respiratory:** dyspnea, acute pulmonary edema, acute respiratory distress syndrome/pneumonitis, interstitial lung disease, interstitial pneumonia, respiratory failure, and pulmonary fibrosis, which may be fatal. Radiation pneumonitis in patients receiving concomitant radiotherapy.
Paclitaxel/Taxol	7038	**BOXED WARNING:** Anaphylaxis and severe hypersensitivity reactions characterized by dyspnea and hypotension requiring treatment, angioedema, and generalized urticaria have occurred in 2 to 4% of patients receiving paclitaxel in clinical trials. **Respiratory:** Interstitial pneumonia, lung fibrosis, and pulmonary embolism have been reported. Radiation pneumonitis has been reported in patients receiving concurrent radiotherapy.Pleural effusion and

		respiratory failure have been reported.
Capecitabine/Xeloda	5978	Not common
Exemestane/Aromasin	4509	Not common
Carboplatin/Paraplatin	4004	Not common
Xgeva/Denosumab	3941	Not common
Vitamin D3/Vitamin D	3514	Not common
Anastrozole/Arimidex	3409	Not common
Levothyroxine/Synthroid/Levothyroxine sodium	3123	Not common
Zoledronic acid/Zometa	3109	Not common
Acetaminophen/Tylenol	2821	Not common
Cyclophosphamide	2678	**Respiratory:** pulmonary veno-occlusive disease, acute respiratory distress syndrome, interstitial lung disease as manifested by respiratory failure (including fatal outcomes), obliterative bronchiolitis, organizing pneumonia, alveolitis allergic, pneumonitis, pulmonary hemorrhage; respiratory distress, pulmonary hypertension, pulmonary edema, pleural effusion, bronchospasm, dyspnea, hypoxia, cough, nasal congestion, nasal discomfort, oropharyngeal pain, rhinorrhea.
Dexamethasone	2674	Not common
Calcium/Calcium carbonate	2437	Not common
Gabapentin/Neurontin	2266	Not commo

Omeprazole	2233	Not common
Furosemide/Lasix	2033	Not common
Ondansetron	1997	Not common
Aspirin	1811	Not common
Lisinopril	1648	Not common
Metformin	1450	Not common
Levothyroxine	1393	Not common
Tamoxifen	1357	Not common
Amlodipine	1349	Not common
Tylenol	1345	Not common
Vinorelbine/Navelbine	1304	**Pulmonary Toxicity and Respiratory Failure:** Dyspnea (shortness of breath) was reported in 3% of patients; it was severe in 2%. Interstitial pulmonary changes were documented.
Ibuprofen	1235	Not common
Epirubicin	1213	Not common
Lorazepam	1202	Not common
Atorvastatin	1124	Not common
Pantoprazole	1100	Not common
Oxycodone	1044	Not common
Vitamin C	1020	Not common
Vitamin B12	986	Not common
Simvastatin	969	Not common
Loperamide	953	Not common
Metoprolol	940	Not common
Sodium chloride	920	Not common
Apixaban/Eliquis	906	Not common
Hydrochlorothiazide	896	Not common
Losartan	857	Not common
Doxorubicin	851	Not common
Potassium chloride	806	Not common
Tramadol	791	Not common
Alprazolam	776	Not common
Prednisone	719	Not common
Metoclopramide	700	Not common
Morphine	661	Not common

Abbreviations: FAERS, FDA Adverse Event Reporting System.

eTable 5. ROR and aROR for novel antineoplastic agents in breast cancer treatment using ILD as the sole PT.

Drugs	Cases	ROR (95% CI)	aROR (95% CI)	*P* value
**FAERS**				
Lapatinib	26	0.49 (0.33-0.71)	0.29 (0.16-0.52)	< 0.0001
Tucatinib	1	0.04 (0.01-0.28)	0.68 (0.09-5.00)	0.70
Trastuzumab	213	1.32 (1.15-1.52)	0.94 (0.66-1.32)	0.71
Pertuzumab	142	4.44 (3.74-5.26)	2.34 (1.59-3.46)	< 0.0001
Trastuzumab emtansine	55	2.21 (1.69-2.90)	1.89 (1.22-2.93)	0.0040
Trastuzumab deruxtecan	300	17.14 (15.04-19.55)	6.88 (4.76-9.96)	< 0.0001
Sacituzumab govitecan	3	0.13 (0.04-0.39)	0.44 (0.06-3.19)	0.42
Pembrolizumab	18	1.12 (0.70-1.78)	0.69 (0.16-2.89)	0.61
Atezolizumab	56	6.77 (5.18-8.86)	1.01 (0.58-1.75)	0.96
Palbociclib	86	0.17 (0.14-0.22)	0.76 (0.50-1.16)	0.20
Ribociclib	43	0.29 (0.22-0.40)	0.54 (0.28-1.03)	0.063
Abemaciclib	123	3.44 (2.86-4.13)	5.02 (3.31-7.63)	< 0.0001
Olaparib	24	3.71 (2.48-5.57)	1.66 (0.38-7.32)	0.50
Alpelisib	6	0.18 (0.08-0.41)	0.53 (0.13-2.17)	0.38
Everolimus	118	1.44 (1.19-1.73)	1.96 (1.28-3.01)	0.0020
**JADER**				
Lapatinib	31	0.17 (0.12-0.25)	0.31 (0.19-0.51)	< 0.0001
Trastuzumab	391	1.59 (1.42-1.78)	1.73 (1.37-2.18)	< 0.0001
Pertuzumab	224	1.76 (1.52-2.04)	1.87 (1.34-2.61)	< 0.0001
Trastuzumab emtansine	99	1.57 (1.27-1.94)	1.51 (1.06-2.14)	0.023
Trastuzumab deruxtecan	131	6.35 (5.14-7.86)	11.28 (8.39-15.16)	< 0.0001
Pembrolizumab	43	0.49 (0.36-0.67)	1.65 (0.72-3.83)	0.24
Atezolizumab	54	1.91 (1.43-2.54)	1.07 (0.59-1.94)	0.83
Palbociclib	81	0.39 (0.31-0.49)	0.99 (0.69-1.42)	0.94
Abemaciclib	24	3.57 (2.27-5.63)	3.33 (1.79-6.19)	< 0.0001
Olaparib	14	0.58 (0.34-0.99)	0.51 (0.19-1.43)	0.20

**Drugs**	**Cases**	**ROR (95% CI)**	**aROR (95% CI)**	***P* value**
Everolimus	649	8.76 (7.88-9.75)	5.48 (3.94-7.64)	< 0.0001

Abbreviations: ROR, reporting odds ratio; aROR, adjusted reporting odds ratio; ILD, interstitial lung disease; PT, preferred term; CI, confidence interval; FAERS, FDA Adverse Event Reporting System; JADER, Japanese Adverse Drug Event Report.

eTable 6. Time to onset and Weibull shape parameters for interstitial lung disease associated with novel antineoplastic agents in FAERS and JADER databases

Drugs	TTO (days): median (IQR)	Scale parameter: alpha (95% CI)	Shape parameter: beta (95% CI)	Type
**FAERS**				
Lapatinib	38.5 (23-82)	62.16 (41.92-82.41)	0.98 (0.74-1.21)	Random failure
Tucatinib	119.5 (41.5-196)	136.47 (17.91-255.03)	0.97 (0.32-1.61)	Random failure
Trastuzumab	90 (39-203)	163.35 (128.49-198.22)	0.77 (0.68-0.86)	Early failure
Pertuzumab	57 (24.5-112)	89.74 (68.47-111.01)	0.84 (0.72-0.97)	Early failure
T-DM1	115.5 (38-211.5)	156.56 (113.04-200.09)	1.01 (0.80-1.22)	Random failure
T-Dxd	85 (33.5-161)	123.21 (99.52-146.91)	1.02 (0.87-1.16)	Random failure
SG	20 (11.8-48)	39.01 (12.83-65.19)	0.89 (0.50-1.28)	Random failure
Pembrolizumab	58.5 (47.2-60.8)	77.64 (45.53-109.74)	1.26 (0.84-1.68)	Random failure
Atezolizumab	71 (22-166)	199.01 (85.26-152.75)	0.88 (0.72-1.04)	Random failure
Palbociclib	138 (74-323)	236.39 (184.06-288.72)	0.86 (0.75-0.98)	Early failure
Ribociclib	76 (28-210)	148.03 (108.77-187.29)	0.83 (0.70-0.96)	Early failure
Abemaciclib	57.5 (27.8-143)	104.98 (77.69-132.27)	0.83 (0.71-0.96)	Early failure
Olaparib	19 (15-250)	71.12 (17.19-125.04)	0.76 (0.44-1.08)	Random failure
Alpelisib	38 (13-164)	88.73 (27.95-149.50)	0.73 (0.46-1.01)	Random failure
Everolimus	56 (23.5-104.5)	87.00 (74.69-99.30)	0.92 (0.84-1.00)	Random failure
**JADER**				
Lapatinib	66 (34.8-170.5)	108.46 (63.58-153.35)	0.95 (0.68-1.21)	Random failure
Trastuzumab	77 (44.5-123.5)	139.17 (111.12-167.22)	0.78 (0.70-0.87)	Early failure
Pertuzumab	72 (36-111.2)	108.93 (78.66-139.19)	0.83 (0.70-0.96)	Early failure
T-DM1	168 (63-297)	232.23 (174.22-290.24)	1.19 (0.93-1.46)	Random failure
T-Dxd	64 (19-189)	102.12 (42.61-161.64)	0.98 (0.55-1.42)	Random failure
Pembrolizumab	61 (23-101)	68.77 (48.77-88.77)	1.72 (1.04-2.40)	Wear-out failure
Atezolizumab	135.5 (54-197)	151.82 (101.23-202.42)	1.09 (0.79-1.39)	Random failure
Palbociclib	138 (42-257)	200.61 (115.02-286.21)	0.93 (0.66-1.21)	Random failure
Abemaciclib	205 (89.8-279)	246.94 (139.91-353.963)	1.27 (0.76-1.79)	Random failure
Olaparib	150 (47-248.5)	154.23 (49.66-258.81)	1.14 (0.42-1.87)	Random failure
Everolimus	61 (44-104.5)	92.82 (82.09-103.55)	1.31 (1.18-1.43)	Random failure

Abbreviations: FAERS, FDA Adverse Event Reporting System; JADER, Japanese Adverse Drug Event Report; TTO, time to onset; IQR, interquartile range; CI, confidence interval; T-DM1, trastuzumab emtansine; T-Dxd, trastuzumab deruxtecan; SG, sacituzumab govitecan.

eTable 7. Cox regression analysis of clinical characteristics on ILD reports in FAERS and JADER

Characteristics	HR (95% CI)	*P* value
**FAERS**		
Age (years)	1.0097 (1.0036–1.0159)	0.0020
Weight (kg)	0.9783 (0.9729–0.9836)	< 0.0001
Number of drugs taken	1.0036 (0.9918–1.0155)	0.55
**JADER**		
Age (years)	1.0183 (1.0094–1.0270)	< 0.0001
Weight (kg)	1.0077 (0.9990–1.0170)	0.081
Number of drugs taken	0.9924 (0.9757–1.0090)	0.38

Abbreviations: ILD, interstitial lung disease; FAERS, FDA Adverse Event Reporting System; JADER, Japanese Adverse Drug Event Report; HR, hazard ratio.
