# Supplementary material for: Disproportionality analysis of interstitial lung disease associated with novel antineoplastic agents during breast cancer treatment: a pharmacovigilance study
Source: eClinicalMedicine. 2025 Mar 18;82:103160. doi: 10.1016/j.eclinm.2025.103160 (PMC11957809; doi:10.1016/j.eclinm.2025.103160)
Supplement: Tracked changes Supplementary [file mmc2.docx]

**Supplementary**

**Additions to the manuscript**

**Methods**

**Statistical analysis**

The following formula was used to calculate the ROR and 95% confidence interval (CI):

$$ROR=\frac{a/c}{b/d}$$

$$95\% CI=e^{\ln\left( ROR \right)\pm1.96\sqrt{\frac{1}{a}+\frac{1}{b}+\frac{1}{c}+\frac{1}{d}}}$$

A represents reports of target events involving target drugs; b represents reports of non-target events involving target drugs; c represents reports of target events involving drugs other than the target drugs; d represents reports of non-target events involving drugs other than the target drugs.

**Results**

**Disproportionality analysis**

In FAERS, we further conducted a signal detection analysis for the top 10 most frequently reported PTs under the SMQ level (Figure 2C). PTs such as interstitial lung disease (ROR 1.23; 95% CI 1.13-1.33), pneumonitis (ROR 1.75; 95% CI 1.60-1.91), pulmonary fibrosis (ROR 1.49; 95% CI 1.21-1.85), pulmonary toxicity (ROR 1.50; 95% CI 1.16-1.95), lung opacity (ROR 3.02; 95% CI 1.98-4.16), and immune-mediated lung disease (ROR 2.78; 95% CI 1.07-7.24) all showed signal of disproportionate reporting of novel antineoplastic agents. Among these, interstitial lung disease (n=1,214) and pneumonitis (n=1,183) accounted for the vast majority of the reports. In the JADER database, we performed disproportionality and sensitivity analyses after excluding cases suspected of being duplicates of FAERS database. A total of 4 novel antineoplastic agents showed a signal of disproportionate reporting for ILD both before and after adjustment: Everolimus [ROR 6.57 (5.88-7.34), aROR 2.49 (1.62-3.84)]; T-Dxd [ROR 4.59 (3.61-5.79), aROR 11.05 (8.07-15.13)]; abemaciclib [ROR 2.37 (1.36-3.90), aROR 2.34 (1.11-4.91)]; T-DM1 [ROR 1.53 (1.24-1.88), aROR 1.62 (1.13-2.33)] (eTable 2).

**Comparison with chemotherapy**

In FAERS, 5 chemotherapeutic drugs were identified as signal of disproportionate reporting for ILD: Eribulin (ROR 4.68; 95% CI 3.97-5.53), 5-fluorouracil (ROR 2.51; 95% CI 1.81-3.47), paclitaxel (ROR 2.19; 95% CI 1.97-2.45), gemcitabine (ROR 2.11; 95% CI 1.59-2.80) and doxorubicin (ROR 1.59; 95% CI 1.28-1.97) (eFigure 4). In JADER, 4 drugs were confirmed: Docetaxel (ROR 1.67; 95% CI 1.45-1.93), paclitaxel (ROR 1.49; 95% CI 1.31-1.69), cyclophosphamide (ROR 1.32; 95% CI 1.15-1.51), and eribulin (ROR 1.24; 95% CI 1.03-1.48) (eFigure 5). It is evident that, from an overall perspective, there are more signals of disproportionate reporting for novel antineoplastic agents compared with conventional chemotherapeutic agents, as shown in the volcano plots from both the FAERS (Figure 5A) and JADER (Figure 5B). Furthermore, in FAERS, univariate logistic regression analysis showed that novel antineoplastic agents were significantly associated with the occurrence of ILD compared to conventional chemotherapeutic agents (OR = 2.47, 95% CI: 2.16-2.81, *P* < 0.0001). Similarly, in JADER, novel antineoplastic agents were also significantly associated with ILD compared to conventional chemotherapeutic agents (OR = 1.61, 95% CI: 1.37-1.88, *P* < 0.0001). These findings suggest stronger reporting associations between novel antineoplastic agents and ILD than those observed with conventional chemotherapeutic agents.

**Factors associated with ILD**

Results of multivariate Cox regression analysis from FAERS indicated that both age and weight were potential factors influencing the occurrence of ILD. Specifically, each additional year was associated with an increase in the odds of reporting ILD by approximately 0.97% (HR = 1.0097, 95% CI: 1.0036-1.0159, *P* = 0.0020), while each additional kilogram of body weight was associated with a decrease in the odds of reporting ILD by approximately 2.17% (HR = 0.9783, 95% CI: 0.9729-0.9836, *P* < 0.0001). In contrast, the number of drugs taken showed no significant impact on ILD (HR = 1.0036, 95% CI: 0.9918-1.0155, *P* = 0.55). The analysis from the JADER database also confirmed the consistency of age as a potential factor, with each additional year increasing the odds of reporting ILD by about 1.83% (HR = 1.0183, 95% CI: 1.0094-1.0270, *P* < 0.0001). However, neither weight (HR = 1.0077, 95% CI: 0.9990-1.0170, *P* = 0.081) nor the number of drugs taken (HR = 0.9924, 95% CI: 0.9757-1.0090, *P* = 0.038) showed statistically significant associations with ILD (eTable 7).

**Disproportionality analysis reported by medical professionals**

In the FAERS and JADER databases, reports are predominantly submitted by healthcare professionals, pharmaceutical companies, and consumers. To enhance data quality and reduce erroneous reports, we excluded cases reported by non-professionals such as consumers and lawyers. Disproportionality analysis was specifically conducted on ILD cases reported by healthcare professionals, including physicians, pharmacists, and nurses. The analysis demonstrated that, regardless of whether it is from the FAERS or JADER database, the drugs associated with ILD as reported by healthcare professionals are generally consistent with those identified in the overall reports by all personnel. (eFigure 6 and eFigure 7).

**Discussion**

We found that novel antineoplastic agents are more strongly associated with ILD compared to traditional chemotherapy drugs, emphasizing the need to be vigilant about potential pulmonary toxicity when using these new drugs. This finding also reminds us that while the efficacy of new drugs is important, it is equally crucial to thoroughly assess and monitor their potential serious adverse effects.

In analyzing potential factors for ILD, both age and weight significantly impact the incidence of ILD. With increasing age, due to the typically weaker immune function and chronic inflammatory states in elderly populations, the odds of ILD also increases. Conversely, an increase in weight is negatively correlated with the odds of ILD. This observation aligns with existing studies where a higher BMI and body weight have been linked to lower mortality rates and slower disease progression, suggesting better nutritional status and greater energy reserves that aid in managing chronic illnesses.^30^ Additionally, higher body weight might be associated with greater lung capacity and enhanced respiratory functions, offering certain protective benefits. The number of concomitant drugs did not significantly impact ILD, likely due to the inclusion of supportive or pre-treatment drugs, which are less likely to induce ILD. Additionally, only ADR reports categorized as “primary suspect” or “suspected medicine” were analysed, focusing on drugs most likely responsible for ILD. This may have reduced the influence of the total drug count on ILD. These findings underscore the necessity of incorporating age and weight considerations into therapeutic strategies for ILD patients to optimize outcomes.

**References**

**40**. Comes A, Wong AW, Fisher JH, et al: Association of BMI and Change in Weight with Mortality in Patients with Fibrotic Interstitial Lung Disease. Chest 161:1320–1329, 2022

**Figure legends**

**eFigure 1. Cox regression analysis of ILD reports across different classes of novel antineoplastic agents in the FAERS database.** Abbreviations: HR, hazard ratio; TKIs, tyrosine kinase inhibitors; mAbs, monoclonal antibodies; ADCs, antibody-drug conjugates; PD-1/PD-L1, programmed death-1/programmed death-ligand 1; CDK4/6, cyclin-dependent kinase 4/6; PARP, poly ADP-ribose polymerase; PI3K/AKT/mTOR, phosphatidylinositol 3-kinase/protein kinase B/mammalian target of rapamycin.

**eFigure 2. Kaplan-Meier analysis of the probability of developing ILD between the drug groups and the control group. TKIs (A), mAbs (B), ADCs (C), ICIs (D), CDK4/6 inhibitors (E), PARP inhibitors (F), PI3K/AKT/mTOR inhibitors (G). Each curve compares the probability of ILD occurrence over time for patients treated with a specific drug class against other drugs.**Abbreviations: HR, hazard ratio; TKIs, tyrosine kinase inhibitors; mAbs, monoclonal antibodies; ADCs, antibody-drug conjugates; PD-1/PD-L1, programmed death-1/programmed death-ligand 1; CDK4/6, cyclin-dependent kinase 4/6; PARP, poly ADP-ribose polymerase; PI3K/AKT/mTOR, phosphatidylinositol 3-kinase/protein kinase B/mammalian target of rapamycin.

**eFigure 3.** **A column plot showing the fatality proportions and number for the different SMQs in the FAERS database.**

Abbreviations: SMQ, standardized MedDRA query; FAERS, FDA Adverse Event Reporting System.

**eFigure 4. Forest plot of ROR values for different chemotherapeutic agents associated with ILD in the FAERS database.**

Abbreviations: ROR, reporting odds ratio; CI, confidence interval; ILD, interstitial lung disease; FAERS, FDA Adverse Event Reporting System.

**eFigure 5. Forest plot of ROR values for different chemotherapeutic agents associated with ILD in the JADER database.**

Abbreviations: ROR, reporting odds ratio; CI, confidence interval; ILD, interstitial lung disease; JADER, Japanese Adverse Drug Event Report.

**eFigure 6. Forest plot of ROR values for different novel antineoplastic agents associated with ILD in the FAERS database, based on reports from healthcare professionals.**

Abbreviations: ROR, reporting odds ratio; CI, confidence interval; ILD, interstitial lung disease; FAERS, FDA Adverse Event Reporting System.

**eFigure 7. Forest plot of ROR values for different novel antineoplastic agents associated with ILD in the JADER database, based on reports from healthcare professionals.**

Abbreviations: ROR, reporting odds ratio; CI, confidence interval; ILD, interstitial lung disease; JADER, Japanese Adverse Drug Event Report.

**eTable 1. The PTs included in the narrow SMQ of ILD**

Abbreviations: PT, preferred term; SMQ, standardized MedDRA query; ILD, interstitial lung disease.

**eTable 2. ROR and aROR for ILD associated with antineoplastic agents in breast cancer treatment using the JADER database after exclusion of suspected duplicate cases.**

Abbreviations: ROR, reporting odds ratio; aROR, adjusted reporting odds ratio; ILD, interstitial lung disease; JADER, Japanese Adverse Drug Event Report; CI, confidence interval.

**eTable 3. Raw data used in the disproportionality analysis to calculate the ROR**

Abbreviations: ROR, reporting odds ratio; a, reports of target events involving target drugs; b, reports of non-target events involving target drugs; c, reports of target events involving drugs other than the target drugs; d, reports of non-target events involving drugs other than the target drugs; FAERS, FDA Adverse Event Reporting System; TKIs, tyrosine kinase inhibitors; mAbs, monoclonal antibodies; ADCs, antibody-drug conjugates; PD-1/PD-L1, programmed death-1/programmed death-ligand 1; CDK4/6, cyclin-dependent kinase 4/6; PARP, poly ADP-ribose polymerase; PI3K/AKT/mTOR, phosphatidylinositol 3-kinase/protein kinase B/mammalian target of rapamycin; JADER, Japanese Adverse Drug Event Report; SMQ, standardized MedDRA query.

**eTable 4. Top 50 concomitant drugs with novel antineoplastic agents in the FAERS database.**

Abbreviations: FAERS, FDA Adverse Event Reporting System.

**eTable 5. ROR and aROR for novel antineoplastic agents in breast cancer treatment using ILD as the sole PT.**

Abbreviations: ROR, reporting odds ratio; aROR, adjusted reporting odds ratio; ILD, interstitial lung disease; PT, preferred term; CI, confidence interval; FAERS, FDA Adverse Event Reporting System; JADER, Japanese Adverse Drug Event Report.

**eTable 6. Time to onset and Weibull shape parameters for interstitial lung disease associated with novel antineoplastic agents in FAERS and JADER databases.**

Abbreviations: FAERS, FDA Adverse Event Reporting System; JADER, Japanese Adverse Drug Event Report; TTO, time to onset; IQR, interquartile range; CI, confidence interval; T-DM1, trastuzumab emtansine; T-Dxd, trastuzumab deruxtecan; SG, sacituzumab govitecan.

**eTable 7. Cox regression analysis of clinical characteristics on ILD reports in FAERS and JADER.**

Abbreviations: ILD, interstitial lung disease; FAERS, FDA Adverse Event Reporting System; JADER, Japanese Adverse Drug Event Report; HR, hazard ratio.
